# Reconciling ice core CO_2_ and land-use change following New World-Old World contact

**DOI:** 10.1038/s41467-024-45894-9

**Published:** 2024-03-05

**Authors:** Amy C. F. King, Thomas K. Bauska, Edward. J. Brook, Mike Kalk, Christoph Nehrbass-Ahles, Eric. W. Wolff, Ivo Strawson, Rachael H. Rhodes, Matthew B. Osman

**Affiliations:** 1 British Antarctic Survey, Cambridge, UK; 2 College of Earth, Ocean and Atmospheric Sciences, Oregon State University, Corvallis, OR USA; 3 Department of Earth Sciences, University of Cambridge, Cambridge, UK; 4 Department of Geography, University of Cambridge, Cambridge, UK; 5 Present Address: National Physical Laboratory, Teddington, UK

**Keywords:** Cryospheric science, Palaeoclimate, Carbon cycle

## Abstract

Ice core records of carbon dioxide (CO_2_) throughout the last 2000 years provide context for the unprecedented anthropogenic rise in atmospheric CO_2_ and insights into global carbon cycle dynamics. Yet the atmospheric history of CO_2_ remains uncertain in some time intervals. Here we present measurements of CO_2_ and methane (CH_4_) in the Skytrain ice core from 1450 to 1700 CE. Results suggest a sudden decrease in CO_2_ around 1610 CE in one widely used record may be an artefact of a small number of anomalously low values. Our analysis supports a more gradual decrease in CO_2_ of 0.5 ppm per decade from 1516 to 1670 CE, with an inferred land carbon sink of 2.6 PgC per decade. This corroborates modelled scenarios of large-scale reorganisation of land use in the Americas following New World-Old World contact, whereas a rapid decrease in CO_2_ at 1610 CE is incompatible with even the most extreme land-use change scenarios.

## Abstract (web summary)

Ice core records of gradually declining atmospheric carbon dioxide (CO_2_) for the period 1450–1700 CE support modelled scenarios of large-scale reorganisation of land use in the Americas following New World-Old World contact.

## Introduction

Ice cores present a unique opportunity to perform direct measurements on ancient atmosphere encapsulated within the ice. Records of the last 2000 years, the Common Era, offer the highest resolution archives of greenhouse gases, allowing us to observe multidecadal to centennial scale atmospheric changes, and providing a baseline for preindustrial atmospheric values. Ice core records of CO_2_ are therefore a powerful tool in understanding the global carbon cycle.

**Fig. 1:**
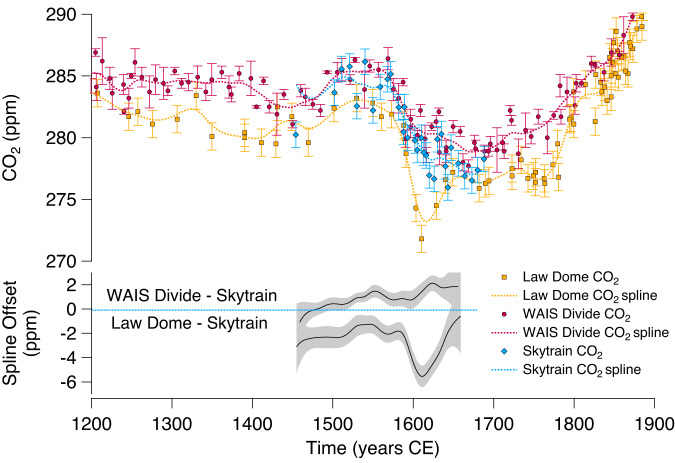
Common Era ice core carbon dioxide (CO_2_) records and their offsets. Currently available high-resolution CO_2_ records shown for the Common Era, from Law Dome^1^ and West Antarctic Ice Sheet (WAIS) Divide^4^ ice cores, and the CO_2_ record from Skytrain (this study) with standard deviation error bars as published for Law Dome and WAIS Divide, or as in methods for Skytrain. Also plotted are generated splines (see methods) and the offsets between splines when referenced to Skytrain, highlighting the distinctive 1610 CE minimum in CO_2_ in the Law Dome record. Grey shading on the offsets is the error of the splines.

Highest-quality CO_2_ records for the last 2000 years are limited to just two archives: the Law Dome and West Antarctic Ice Sheet (WAIS) Divide ice cores^1–4^ (Fig. 1). Much lower resolution records are also available and have been used to confirm broader past-millennium CO_2_ trends, but application to studies of high-resolution changes, as in this study, is limited due to their significant signal attenuation^5,6^. Each ice core has limitations in terms of accurately recording atmospheric variability. The Law Dome ice core offers the highest accumulation-rate (60 cm water eq. yr^−1^) and therefore potential for the highest temporal resolution gas record preserved in the ice (gas age distribution width of 8 years at Full Width Half Maximum (FWHM)) due to minimal smoothing by gas diffusion and mixing in the firn column prior to bubble close-off. The Law Dome data were measured with pioneering techniques in the 1990s, greatly advancing our understanding of CO_2_ in the last millennium^7^. However, the sampling resolution is relatively low in some periods. The dataset has subsequently undergone multiple revisions, filling data gaps, extending the record, investigating differences between early and later measurements, and adjusting error values^2,8^. The WAIS Divide record provides higher sample resolution and precision, but the core has a lower accumulation rate (19.8 cm water eq. yr^−1^), which results in greater smoothing of the gas record by firn-based processes (gas age distribution width of 19 years FWHM). There are multidecadal features present in the Law Dome record that are not observed in WAIS Divide. This may be partly due to the greater extent of firn-based smoothing at WAIS Divide but, even so, the most rapid CO_2_ changes present in the Law Dome record have been difficult to explain within our current understanding of the carbon cycle and in even the most complex Earth System Models^9–11^. In addition to this uncertainty regarding atmospheric CO_2_ history, the absolute mixing ratios of WAIS Divide range from 0 to 6 ppm higher than those of Law Dome between 750 and 1800 CE^3,4^, though the exact cause of this artefact remains elusive^2,3^.

The Law Dome and WAIS Divide ice core records of CO_2_ are fundamental to modelling studies of climate and carbon cycling throughout the Common Era^4,5,12^. The Law Dome CO_2_ record is used exclusively in the Paleoclimate Modelling Intercomparison Project^13,14^ which contributes to climate model evaluation at the IPCC level^15^. Several rapid CO_2_ changes in the Law Dome record have been highlighted as potentially coinciding with major events in history, such as plagues, migrations, and wars^16–18^, as well as inferred climate-CO_2_ feedback^5^. The most high-profile example is that of the distinctive Law Dome 1610 CE CO_2_ minimum, a rapid decrease in CO_2_ of ~10 ppm over 90 years, with a distinct minimum at 1610 CE (Fig. 1). One hypothesis is that the distinctive low value (termed the Orbis Spike^18^) is caused by the contact between New World and Old World populations. Subsequent pandemic-driven population decrease, followed by large-scale land abandonment in the Americas, could have driven land biosphere regrowth and CO_2_ uptake^18–20^. This interpretation has led to the Law Dome 1610 CE CO_2_ minimum being suggested as a marker for the start of the Anthropocene^18^. However, the WAIS Divide CO_2_ record does not exhibit a 1610 CO_2_ minimum and instead shows a smaller and more gradual reduction in CO_2_ into the 17th century (Fig. 1).

The observed differences in current records create significant questions in our carbon cycle interpretation for the time. Here we directly address this challenge by presenting a high-resolution CO_2_ record through the period of interest from the Skytrain Ice Rise (Skytrain) ice core^21,22^.

## Results and discussion

### Skytrain ice core CO_2_ record

In this study we conducted measurements of CO_2_ at 31 depth intervals spanning the period 1454 to 1688 CE in the Skytrain ice core. Samples were measured using the Oregon State University (OSU) crusher system^3^ with 2–3 replicates per sample giving a pooled standard deviation of 1.0 ppm. Smoothing splines were generated for each archive record using a bootstrapped Monte Carlo simulated cubic smoothing spline procedure (*n* = 10,000 histories with a 50-year cut-off). The splines allow clearer comparison of the trends of the records.

The Skytrain record shows a gradual decrease in CO_2_ into the 17th century (Fig. 1), with a reduction in CO_2_ of 8.0 ppm over 157 years (Table 1) based on the spline fit to the data. This does not agree with the greater, more rapid decrease in the Law Dome records, which indicate a total reduction in CO_2_ of 9.9 ppm over 84 years. The Skytrain record instead aligns with the previous WAIS Divide record which shows a total decrease in CO_2_ of 6.7 ppm over 117 years. Average decadal rates of change in atmospheric CO_2_ across the period of declining values emphasise differences between records, from 1.2 ppm per decade for Law Dome to 0.6 ppm per decade at Skytrain and 0.5 ppm at WAIS Divide. Interestingly, the absolute CO_2_ values of Skytrain lie between those of the Law Dome and WAIS Divide records, but with some periods of large intra-core difference that cannot be atmospheric in origin, for example between 1525 CE and 1575 CE.

**Table 1:** Summary of spline-based CO_2_ changes through the 17th century decrease for the Skytrain, Law Dome and West Antarctic Ice Sheet (WAIS) Divide ice cores, and a data compilation of the three records

	CO_2_ max (ppm)	Max year (CE)	CO_2_ min (ppm)	Min year (CE)	CO_2_ decrease (ppm)	Decrease duration (Years)	Decadal rate of decrease (ppm)
Skytrain	285.0 ± 1.2	1515	277.0 ± 1.7	1672	8.0	157	0.5
Law Dome	283.1 ± 1.2	1532	273.2 ± 1.7	1616	9.9	84	1.2
WAIS Divide	285.5 ± 0.7	1555	278.8 ± 0.5	1672	6.7	117	0.6
Compilation	284.4 ± 0.4	1532	277.6 ± 0.3	1673	6.8	141	0.5

### Firn-based smoothing of the ice core gas records

A caveat to consider when comparing ice core gas records is that each record has undergone different levels of smoothing (i.e., low pass filtering) in the firn and during bubble close off. The signal attenuation for Law Dome is smaller (accumulation 60 cm water eq. yr^−1^; gas age distribution 8 yr FWHM) than the WAIS Divide record (accumulation 19.8 cm water eq. yr^−1^; gas age distribution 19 yr FWHM) and Skytrain record (accumulation 13.5 cm water eq. yr^−1^; gas age distribution ~ 27 yr FWHM). It is thus expected that Law Dome captures faster rates of change and higher frequency variability. But is it possible that the broad minimum around 1610 CE (about 40 years) was an atmospheric CO_2_ change that was recorded at Law Dome but completely smoothed away at both WAIS Divide and Skytrain? To answer this, we take a multi-stage approach.

**Fig. 2:**
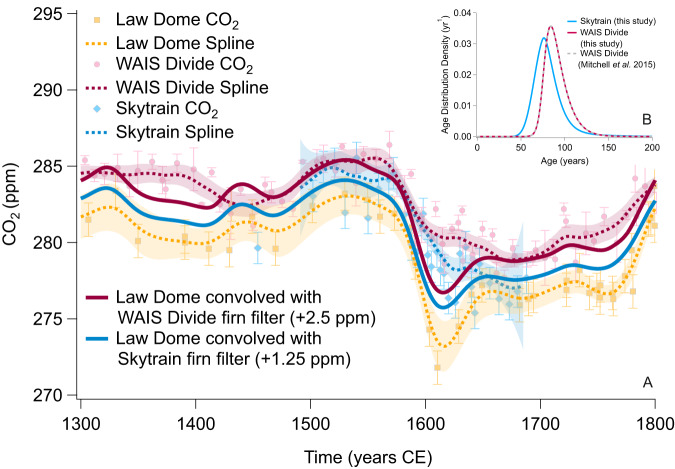
Observing effects of firn smoothing on the carbon dioxide (CO_2_) records using convolution of records with firn filters. **A** Convolutions of modelled firn filters for the West Antarctic Ice Sheet (WAIS) Divide and Skytrain cores with the raw Law Dome CO_2_ record, overlying the three original records and their splines, as in Fig. 1, and the 1-sigma bootstrapped confidence intervals for comparison. Each convolution has a correction applied to the absolute values to account for the offset between Law Dome and WAIS Divide or Skytrain, to aid visual comparison to original records. **B** Firn filters generated in this study for the WAIS Divide and Skytrain cores, representing the firn smoothing of the atmospheric gas record for each ice core site, alongside a previously published WAIS Divide filter that includes a stochastic model of bubble closure to mimic layering to show reproducibility.

Firstly, we assume the Law Dome record, although undergoing some smoothing, is a close representation of the true atmospheric history, given the high accumulation rate of the site. We then convolve the Law Dome record with the firn filters of WAIS Divide and Skytrain. These firn filters, or age distributions, were generated using the Oregon State University (OSU) firn model (see methods) and model the firn smoothing specific to each ice core site^23–25^. The resultant convolved histories should closely reproduce the WAIS Divide and Skytrain records. This is not the case (Fig. 2). Instead, the convolutions preserve a distinctive 1610 CE CO_2_ minimum whereas this is not present in the WAIS Divide and Skytrain records.

We note here that the Law Dome CO_2_ record has itself experienced some smoothing in the firn, so while close to the atmospheric record, it is not a true representation. It could be possible to deconvolve Law Dome with a modelled reverse filter to the original signal^5^. However, the 1610 CE CO_2_ minimum in the Law Dome record would only be sharper still, and thus the convolutions would also then show a stronger 1610 CE CO_2_ minimum, something which only disagrees more strongly with the data. Using the original record alone avoids any potential errors from the accuracy of the modelled reverse filter being introduced.

The convolution predicts that the 1610 CE CO_2_ minimum should be preserved in the WAIS Divide and Skytrain cores if it is a true paleo-atmospheric signal, assuming no unexpected changes have occurred, but could our firn filters be underestimating the degree of smoothing in the cores?^44^ For example, our firn model does not account for small-scale (cm-scale) density variations from layering and associated effects on bubble closure which can drive some pores to close-off above the traditional lock-in zone and thus lead to age-reversals and slightly broader firn age distributions.

**Fig. 3:**
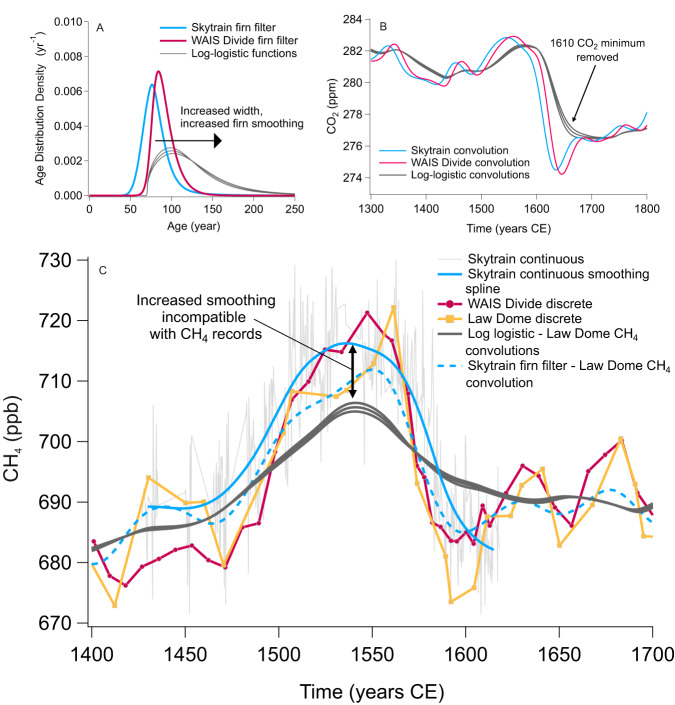
Artificially enhanced smoothing of the ice core records of carbon dioxide (CO_2_) showing incompatibility with the ice core records of methane (CH_4_). **A** West Antarctic Ice Sheet (WAIS) Divide, Skytrain firn filters and log-logistic functions of increased smoothing; **B** convolutions of each firn-filter/function through the Law Dome CO_2_ data, indicating the required level of smoothing for removal of the 1610 CE CO_2_ minimum, i.e., mostly closely recreating the WAIS Divide and Skytrain records (See Methods and Supplementary Information); **C** log-logistic functions convolved with the Law Dome CH_4_ record, demonstrating that the necessary firn smoothing to eliminate the CO_2_ minimum is inconsistent with the observed CH_4_ data.

Lacking an adequate physical model to simulate the potential for greater degrees of firn smoothing we rely on an empirical, independent check of firn smoothing using methane (CH_4_) records. First, we determine whether the 1610 CE CO_2_ minimum seen in the WAIS Divide and Skytrain convolutions can be eliminated by varying the firn filters within a reasonable range. We increase the width of the smoothing until the convolution output best matches the measured CO_2_ records of WAIS Divide and Skytrain using a test which calculates the offset between the convolutions and datasets (See Supplementary Information and Fig. S1). To then test if these are realistic firn filters, we then apply the same firn filters to the CH_4_ records from the three cores, as CH_4_ shows significant multidecadal variability over this time interval that are at least as rapid, if not more rapid, than the CO_2_ variations^26,27^. Convolution of these wider filters with the Law Dome CH_4_ record should recreate the Skytrain and WAIS Divide CH_4_. Instead, the output is significantly more smoothed than the record (Fig. 3) with the rates of change reduced to about 50% (see Supplementary Information). We therefore conclude that no plausible smoothing scenarios exist to account for the absence of the 1610 CE CO_2_ minimum in the WAIS Divide and Skytrain CO_2_ records, and a plausible explanation is that the Law Dome 1610 CE CO_2_ minimum is formed of a small number of artefact data points. The cause of such an artefact is currently unknown but may be related to imperfect post-coring storage conditions^2^.

In an alternative approach, one could use the CH_4_ records from all three cores to determine age distributions of WAIS Divide and Skytrain independently of the CO_2_ record and provide not only estimates of the upper limits of the firn age widths (as done here), but also the lower limits. However, this would require high-resolution, continuous CH_4_ data from Law Dome. Tentatively, we can place some constraints on the lower limit of the firn age distributions by examining a CH_4_ minimum that immediately precedes the CO_2_ drop (~1590 CE). This minimum is fortuitously nearly coincident with the CO_2_ drop of interest (Fig. S4), about half the width of the CO_2_ drop (~20 years versus ~40 years), and about same width as the gas age distribution of Skytrain and WAIS Divide. If our original estimates of smoothing based on firn models were accurate, we would predict that the CH_4_ minimum would be absent in WAIS Divide and Skytrain. This is clearly the case in WAIS Divide record and most likely the case in Skytrain record (although our measurement unfortunately did not extend to the predicted levelling off in CH_4_ post 1600 CE (Fig. 3)). However, other features present in the low-resolution Law Dome data are not obviously comparable to the WAIS Divide and Skytrain features such as divergence in the preceding CH_4_ bump of the 1500’s CE. Measurements of CH_4_ and other gases at the Law Dome site via continuous flow analysis (CFA) are thus a crucial prerequisite for future work on understanding the smoothing imparted on ice core gas records by firn processes.

### Implications for land carbon fluxes

We now explore the biogeochemical implications of the gradual CO_2_ decrease around 1600 CE that our revised CO_2_ history suggests, relative to the rapid 1610 CE CO_2_ minimum seen only in Law Dome.

We performed single deconvolution experiments to reconstruct the net carbon flux to the atmosphere from an external reservoir—in this case the land biosphere. In brief, the atmospheric history of CO_2_ is reproduced in a carbon cycle model by making small adjustments to the land-atmosphere carbon flux at each timestep^28^. The ocean-atmosphere carbon flux (Fig. S5) is allowed to evolve freely and thus acts as either a passive sink or source depending on whether atmospheric CO_2_ is increasing or decreasing, respectively. This technique requires a continuous record of atmospheric CO_2_, interpolated to the model timestep, which we take from the suite of bootstrapped spline fits. We perform the simulation with the OSU Carbon Cycle Box Model^29^ with a 5000 year spin up under pre-industrial conditions and timestep of 0.25 years.

**Fig. 4:**
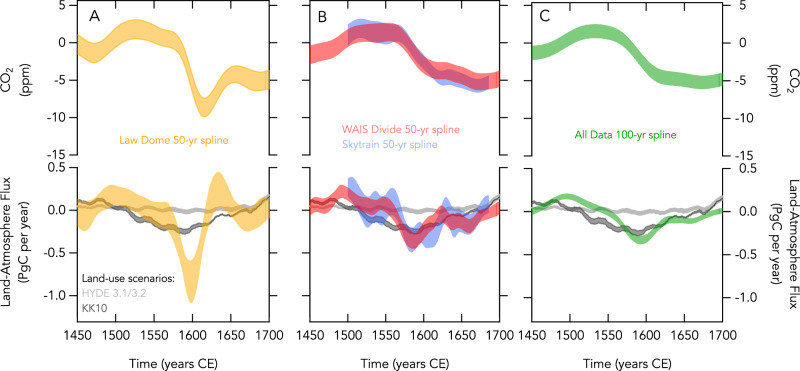
Results from the deconvolution experiments for the various ice core records showing the change in carbon dioxide (CO_2_) and the inferred land-atmosphere carbon flux. **A** The Law Dome-based reconstruction (yellow) with a 50-year spline cut-off. **B** The West Antarctic Ice Sheet (WAIS) Divide (red) and Skytrain-based (blue) reconstructions both with a 50-year spline cut-off. **C** The combined datasets with CO_2_ offsets removed and a 100-year spline cut-off. Across all three panels are a range of scenarios for land-use change and the modelled land-atmosphere carbon flux with the high-end (KK10-based scenarios) highlighted in dark grey and the low-end (Hyde 3.1/3.2-based scenarios) in light grey.

The Law Dome-based reconstructions show a sharp net sink from 1570 to 1620 CE (mean ± 1-sigma s.d. = 0.47 ± 0.19 PgC per year) that strengthens to a maximum up to 1 PgC per year from 1590 to 1600 CE (Fig. 4a). This is followed by a net source of about +0.25 PgC per year from 1620 to 1650 CE. Conversely, the WAIS Divide and Skytrain-based reconstructions (Fig. 4b), which are both in good agreement on timescales longer than a decade, show a much shallower sink from 1570 to 1620 CE (WAIS Divide: 0.24 ± 0.10 PgC per year; Skytrain: 0.28 ± 0.13 PgC per year). A compilation that combines all the data with a more conservative, 100-year spline cut-off period (See Supplementary Information) yields a similarly shallow sink with a slightly tighter constraint (all data: 0.26 ± 0.05 PgC per year) (Fig. 4c). This is in broad agreement with a previous study which utilised a double deconvolution technique that simultaneously constrained the land and ocean fluxes using both the concentration and isotopic composition of CO_2_ from WAIS Divide^4^. Over a slightly broader minimum from 1550 to 1650 CE, the double deconvolution yielded a mean land carbon sink of 0.34 ± 0.06 PgC per year. The full model output for each ice core record is available in Supplementary Data File 2.

We now compare our top-down estimates from the three ice cores with bottom-up scenarios for anthropogenic land-use change (Fig. 4). The bottom-up scenarios can broadly be divided into two groups: low-end scenarios that are driven by HYDE 3.1 and 3.2 land use datasets^30^ and high-end scenarios driven by the KK10 land use^20^. Both scenarios are driven by similar changes in population but differ significantly in their assumed per-capita land use with the high-end scenarios averaging 7 hectares per capita (but varying with population density) and the low-end scenarios set at a constant 0.5 hectares per capita^20,31^. This results in much greater areas of land abandonment and subsequent regrowth of natural terrestrial biomass in the New World in the high-end scenarios. The details of the scenarios, which utilised the LPX Dynamic Global Vegetation Model, are described in Stocker et al., 2017. Note that net uptake by peatlands is relatively minor on the shorter timescale of interest here (0.03–0.05 PgC per year)^4,31^.

The low-end land use scenarios, which show negligible variability in land carbon uptake during the period considered, are inconsistent with all top-down reconstructions. In contrast, the high-end scenarios are in good agreement with the WAIS Divide, Skytrain and combined reconstruction, but are inconsistent with the Law Dome reconstruction. This supports our analysis that the 1610 CE CO_2_ minimum is implausibly large and reinforces previous modelling work that showed the timescale for major regrowth is too long to induce a dramatic dip in atmospheric CO_2_^9^. The more gradual uptake we estimate (~0.26 PgC per year) suggests the high-end scenarios are sufficient to explain the drop in CO_2_. This challenges previous work which, based on the longer-term carbon balance over the last millennium^4^, suggested the high-end scenarios were implausible, as well as recent work which revised downward the per-capita land use in the Americas^11^.

It is important to note that underlying the state-of-the art land use and carbon cycle models are population estimates that are very coarse resolution (100 years until 1700 CE). Now that our data precisely define the timing and magnitude of carbon uptake, further work modelling population dynamics, land-use change and carbon cycle changes during the New World Pandemics is warranted.

Alternatively, the CO_2_ decrease could be due in some part to natural carbon cycle feedbacks that were triggered by a cooling in the Northern Hemisphere^5^. In the case of the 1610 CE CO_2_ minimum this would require a sudden cooling and similarly rapid warming. Intriguingly, many tree-ring based reconstructions of Northern Hemisphere temperature record a strong cooling around 1600 CE, but this oscillation is not particularly exceptional in the wider context of decadal-scale cold periods of the last millennium. For context, the two coldest decades of the last millennium are in the 1800s followed by 1462–1471, 1695–1704 and 1452–1461 as the 3rd, 4th and 5th coldest, respectively^32^. Thus, if the terrestrial carbon cycle was particularly sensitive to rapid coolings, most crucially, with a response time capable of driving a decadal scale drop in CO_2_ as observed in the Law Dome ice core, we would expect similarly large CO_2_ decreases following 1450/60 s and the early 1700s. Instead, CO_2_ is either stable or even increasing during these intervals. More likely, a series of coolings starting as early as the 1300’s in the Northern Hemisphere (in particular the Arctic) contributed to a gradual uptake of carbon on land and no exceptional carbon cycle feedback is required around 1610 CE.

### Remaining questions

Is the 1610 CE minimum in CO_2_ a natural expression of carbon cycle feedbacks, a global marker of land abandonment, or an artefact of ice core data? Based on data from the Skytrain ice core our analysis provides evidence that the large 1610 CE CO_2_ minimum is not a true atmospheric signal, and that other, lower accumulation ice core records are faithfully recording a gradual atmospheric CO_2_ decrease. The gradual decrease observed in the WAIS Divide and now Skytrain ice cores agrees with our current understanding of climate-carbon feedbacks and high-end scenarios of carbon uptake following land abandonment while a large, sudden dip in atmospheric CO_2_ as in the Law Dome record is inconsistent with all current land-use change scenarios. Whether the CO_2_ decrease resulted from natural or anthropogenic processes, or a combination of the two, remains an open question. It is possible that large-scale land abandonment, in tandem with a hitherto unidentified climate-carbon feedback, could explain the minimum in the Law Dome record.

Furthermore, our evidence against the 1610 CE minimum is indirect and the origin of the low values that make up the minimum in the Law Dome data remains unclear. Remeasurement of existing Law Dome samples or, better yet, collection of new very-high resolution cores in other regions of Antarctica, may improve our understanding of the origin of the feature. With the advancement of measurement techniques of CO_2_ and its isotopes since the first pioneering Law Dome measurements, new records could greatly enhance our understanding of rapid anthropogenic and natural carbon cycle changes.

## Methods

The Skytrain ice core was drilled on Skytrain Ice Rise in West Antarctica in 2018/2019^21^, to a depth of 651 m, reaching ice ages from the last interglacial at its base. The samples in this study are taken between 83.2 m and 104.0 m, with respective ice ages between 1182 CE and 1443 CE^22^, and respective gas ages between 1454 CE and 1688 CE^33^. Accumulation rate in this section is 13.5 cm w.e. yr ^−1^ on average with a present day 10 m temperature of −25.9 ^°^C. For further detailed information on drilling, site characteristics and age scale generation please see references ^21,22,33^.

### Sample dating

Discrete samples throughout the period 950-1750 CE had been previously measured for CH_4_ at approximately 20 yr resolution. Samples were measured using the Oregon State University (OSU) melt-refreeze wet extraction system with associated gas chromatography (GC)^26,34,35^, referenced to the WMO X2004A reference scale^36^ and calibrated using a NOAA primary air standard. An average blank correction of 8.3 ± 2.7 SD ppb, and solubility correction of 1 ppb, have been applied. Uncertainty of the measurements is 2–3 ppb^22^. These measurements were incorporated in to the ST22 chronology^22,33^ for the Skytrain ice core, which is used for all ice and gas depth-age dating in this study. Specifically, we use the ST22-WD ages, as opposed to the ST22-AICC ages.

### CO_2_ analysis

CO_2_ analysis was carried out on the OSU needle crusher extraction device with associated GC analysis^3^. The extraction system composes a vacuum chamber inside which a ~ 2 × 2 × 2 cm ice sample is placed. A pneumatically driven bellows, with head attachment of multiple metal needles, impacts the ice ten times to mechanically crush the sample, releasing the gas trapped inside the ice. This gas is dried inside the vacuum system by flowing it through a U-shaped metal trap cooled to −90 °C using an ethanol-liquid nitrogen slush bath. The remaining gas sample is frozen inside a manifold of sample tubes, kept at −260 °C using a helium compressor. Sample tubes are sealed, transferred to a GC, and warmed to room temperature before injection and analysis.

Measured CO_2_ values are calibrated using a NOAA primary air standard (WMO scale X2019) and adjusted for an average full system blank value of 0.8 ppm. A gravitational correction was applied per-sample based on a measured δ^15^N value of 0.16 per mil^33^, equating to an average correction of 0.7 ppm.

Dependent on the size of the original sample, which was based on availability of ice at the required depth, 2 or 3 replicates were run of either true depth (exact depth replicates located next to each other across the width of the core) or proximal depth (located directly above/below each other in the core). The pooled standard deviation is 1.0 ppm on the final CO_2_ values, with no significant difference in pooled standard deviation observed between the two replicate types. The CO_2_ record, ages and errors are presented in Supplementary Data File 1.

### Continuous CH_4_ analysis

16.5 metres of ice from Skytrain was measured for continuous CH_4_ throughout 1426 to 1617 CE. Samples were measured over a single day using the Continuous Flow Analysis (CFA) system at BAS^22^. Sample gas was extracted from a continuous steam of melt by applying a pressure decrease across an Idex Transfer Line Degasser. The sample was then dehumidified in a Nafion dryer before continuous injection into a SARA (Spectroscopy by Amplified Resonant Absorption) analyser^37,38^, where concentration data are acquired at 4 Hz. Gas measurements were bracketed by calibration routines using NOAA primary air standards to place data on the WMO X2004A reference scale^36^ and to correct for the dissolution of CH_4_ in the melted ice stream^27^. Average CH_4_ dissolution was 11.7 ± 0.5 % and a correction factor of 1.13 is applied to all data. Data presented in this study are binned at 30 s intervals and the bins have a pooled relative standard deviation of 0.9% (1σ), equivalent to an average 6.3 ppb. Over the section analysed, a sum total 3.8 m was unavailable due to previous sampling requirements, compounding gaps in the data relating to ice quality (ca. 3 breaks per metre). The CH_4_ record, ages and errors are presented in Supplementary Data File 1.

### Smoothing splines

Smoothing splines for each CO_2_ record, the Skytrain continuous CH_4_ record, and our final CO_2_ compilation were generated using a bootstrapped Monte Carlo simulated cubic smoothing spline procedure. We applied a random sampling with replacement bootstrap over 10,000 iterations of our dataset. For each iteration, the MATLAB cubic smoothing spline function, ‘csaps’, generates a spline based on the input data, x and y, and the smoothing parameter, p. The smoothing parameter is sensitive to the input records’ time-resolution (e.g., 13 years at Law Dome), so an individual parameter is tuned for each record to produce a spline at half height of the amplitude of a generic cosine test function. Cut-off frequencies for the individual records were set at 50 years, in the mid-range of previous studies^2,39^. This value allows preservation of distinct features in the records, such as the 1610 CE minimum in the CO_2_ records, while not being overly affected by sample variation from intrinsic measurement errors. A longer frequency of 100 years is used for the spline based on a compilation of the datasets. The final spline for each record is a mean of these 10,000 iterations and the spline error, or confidence interval, is the standard deviation of the iterations. Spline parameters are summarised in Table S1. Smoothing splines for each ice core record are presented in Supplementary Data File 1.

### Firn filter modelling

We use the Oregon State University Firn Air Model to generate firn filters for the WAIS Divide and Skytrain ice cores^40–42^. These may also be seen referred to as age distributions and/or Greens Functions in similar studies. Broadly, the model allows input of the individual site characteristics of accumulation rate, site temperature, atmospheric pressure, and snow densities at surface and close-off for a choice of gases and over a specified time, calling the Herron-Langway^43^ model for densification. The model incorporates physical processes of diffusion, advection, mixing, bubble closure and bubble compaction. The model has been validated using reproduction of Greenland firn air measurements and intercomparison with multiple other firn models shows good agreement in their depiction of smoothing under the same conditions^40^. For specific details on parameterisations please see references ^40–42^. Additionally, the model firn filters are sensitive to the assumed diffusivity parameter for the gas of interest (in this case CO_2_). We adopt a previously used approach^41^ which assumes a value for CO_2_ diffusivity of 0.10 cm^2^/s. We note that these firn-filters - the starting point for our enhanced smoothing experiments - are not trained on data, they are purely the consequence of our current, best knowledge of firn smoothing. To check validity of these parameters for our sites, we compare our WAIS Divide firn filter to the previously published WAIS Divide filter^23^, which was generated using the Centre for Ice and Climate (CIC), University of Copenhagen, firn air transport model. The CIC firn filter accounts for more complex processes of bubble closure, for example due to layering, in comparison to the OSU model which more simply defines bubble closure following the rate of bulk firn compaction^41^. Despite these differences the two firn filters very closely agree (Fig. 2b), helping to validate our firn filters and indicating that differences in parameterisations between firn models are not impacting our results. The same diffusivity is thus assumed for Skytrain.

Log-logistic distributions (у) can also be used to represent ice core firn filters ^45^using the equation:

1 $$y=((\beta /\alpha ){(x/\alpha )}^{(\beta -1)})/{(1+{(x/\alpha )}^{\beta })}^{2}$$

which requires the inputs of gas age, $x$, distribution width, $\alpha$, and distribution shape, $\beta$. Other distributions can be used, for example log-normal distributions^24,25^, but a comparative study found them to give virtually the same results while log-logistic functions were the most mathematically tractable^45^. Our firn smoothing experiments require an artificially enhanced amount of smoothing for the WAIS Divide and Skytrain distributions (See ‘Results and discussion’ Fig. 3) to eliminate the 1610 CO_2_ minimum from the Law Dome record during convolution. We firstly tuned the width and shape parameters to create a single function that best represents both the WAIS Divide and Skytrain firn filters. From here the shape parameter value is maintained while applying increasing values of width to force a function of increased firn smoothing. Functions are terminated at three times the median age of the distribution to prevent very long tails^44,45^. The start points of the generated functions are also offset by 75 yrs to match the WAIS Divide and Skytrain model firn filters. Repeating the convolution of the Law Dome CO_2_ record with the artificial firn filters allows us to identify the level of firn smoothing required to eliminate the CO_2_ minimum from the Law Dome record, and better reproduce the WAIS Divide and Skytrain CO_2_ records (Fig. 3b). Three filters are chosen which best reproduce these records, which were the three filters calculated to have the lowest offset to the CO_2_ splines of WAIS Divide and Skytrain (see Supplementary Information and Fig. S1).

One thing to consider is that we are using firn filters generated for the diffusion of CO_2_ in our ice core records, but the diffusion of CH_4_ through the firn column will have a slightly different behaviour and therefore diffusivity parameter, potentially increasing the firn smoothing (CH_4_ diffusivity = 1.367 * CO_2_ diffusivity). We test the effect of this by generating a firn filter for Skytrain using the same OSU Firn Air Model and parameters as previously and changing only the diffusion parameter from that of CO_2_ to that of CH_4_. We repeat the convolution process using this filter and the Law Dome CH_4_ record. Comparing each of the new and previous filters and convolutions shows negligible differences (Fig. S2). Each of our generated firn filters are presented in Supplementary Data File 1.

### Convolutions

All convolutions use MATLAB ‘conv’ where an input function, the firn filter, is convolved with an input time series, the ice core record. The convolution requires that the filter functions are normalised to a sum of one, and functions and time series are of matching time resolution. This is 0.2 yrs, the same as the output of the OSU Firn Model. CO_2_ records are interpolated linearly to this time step preceding convolution.

### Compilation CO_2_ record

To account for the various offsets in the final compilation, we adjust each of the Law Dome and WAIS Divide records in the direction of their average spline offsets to the Skytrain spline, as in Fig. 1. For the Law Dome—Skytrain offset, data through the 1610 CE CO_2_ minimum are excluded from the average offset so as not to artificially enlarge the average offset, which equates to the exclusion of three data points. The resulting adjustment for all Law Dome data is +1.4 ppm, and for WAIS Divide is −1.3 ppm. The final compilation spline is generated using a compilation CO_2_ dataset incorporating the original Skytrain CO_2_ values and the adjusted values for Law Dome and WAIS Divide, using the same bootstrapped cubic smoothing spline as previously but with an increased cut-off frequency of 100 years (Fig. S3).

### Supplementary information

Supplementary Information

Peer Review File

Description of Additional Supplementary Files

Supplementary Dataset 1

Supplementary Dataset 2

## Data Availability

Measurements of CO_2_ and CH_4_ in the Skytrain ice core, gas record smoothed splines, generated firn filters and outputs of the land carbon flux model are provided in the Supplementary Information as Supplementary Data files. Law Dome ice core data used in this study is available from 10.1029/2006GL026152 in the Supporting Information and WAIS Divide ice core data used in this study from 10.1038/ngeo2422 in the Supplementary Information.

## Code availability

The Matlab code for generation of bootstrapped splines and confidence intervals is available on Zenodo, 10.5281/zenodo.10203638.
