# Supplementary material for: Reconciling ice core CO2 and land-use change following New World-Old World contact
Source: Nat Commun. 2024 Mar 5;15:1735. doi: 10.1038/s41467-024-45894-9 (PMC10915154; doi:10.1038/s41467-024-45894-9)
Supplement: Supplementary file 1 — Supplementary Information [file 41467_2024_45894_MOESM1_ESM.pdf]

Supplementary Information for:

Reconciling ice core CO2 and land-use change following New World-Old World contact

Amy C.F. King[1*]; Thomas K. Bauska[1]; Edward. J. Brook[2]; Mike Kalk[2]; Christoph Nehrbass-Ahles[3,†]; Eric Wolff [3]; Ivo Strawson[1,3]; Rachael H. Rhodes[3]; Matthew B. Osman[4]

[1]British Antarctic Survey, Cambridge, UK.
[2]College of Earth, Ocean and Atmospheric Sciences, Oregon State University, Corvallis, Oregon, USA.
[3]Department of Earth Sciences, University of Cambridge, Cambridge, UK.
[4]Department of Geography, University of Cambridge, Cambridge, UK.
[†]Now at National Physical Laboratory, Teddington, UK.
*Corresponding author (amyking@bas.ac.uk)

Choice of increased smoothing log-logistic functions

Our firn smoothing experiments require an artificially enhanced amount of smoothing for the WAIS Divide and Skytrain distributions (See ‘Results and Discussion’ Figure 3) to eliminate the 1610 CO_2 minimum from the Law Dome record during convolution. From our artificially generated filters (see manuscript methods), three filters are chosen which best reproduce these records, which were the three filters calculated to have the lowest offset to the CO_2 splines of WAIS Divide and Skytrain. This was calculated over the specific time window covering the CO_2 drop (Figure S1A). The average offset for each spline and function is then plotted to find the optimal functions, where the offset is lowest (Figure S1B). The different functions are represented by their ‘ɑ’ value, which is the parameter of the log-logistic function that controls the width of the function and therefore the degree of smoothing. For Skytrain, the three best-fit functions, representing a range of options since none are perfect, were ɑ75, ɑ80 and ɑ85. For WAIS Divide the best-fit functions were ɑ70, ɑ75 and ɑ80, a close agreement. There is little difference between these two options, so we choose to take forward the range for Skytrain based on our later experiments being used to investigate other Skytrain gas records specifically, though our conclusions are not impacted either way.

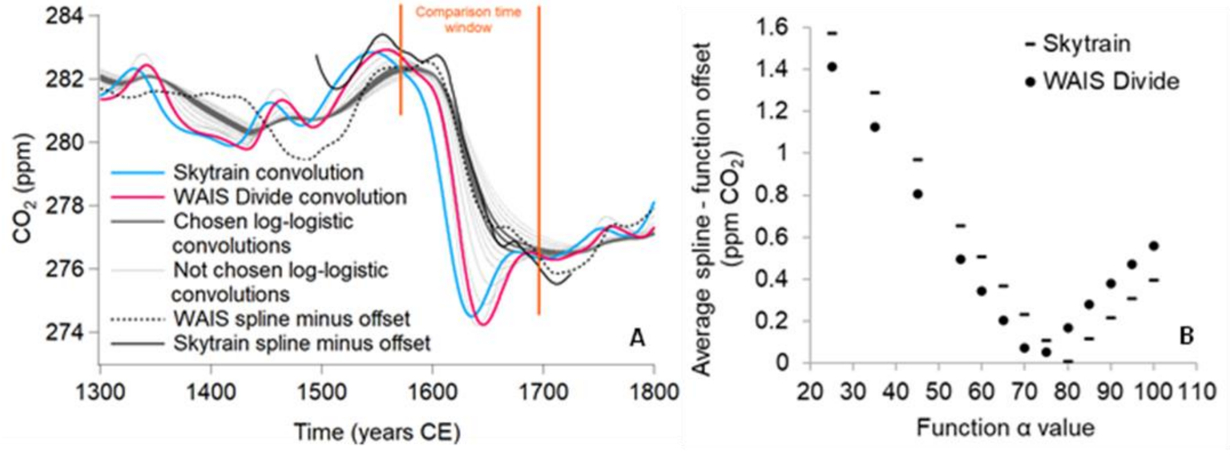

Figure S1: Choice of log-logistic functions which best recreate the Skytrain and WAIS Divide CO_2 records (i.e. which eliminate the '1610 dip' when convolved with the Law Dome CO_2 record) for enhanced smoothing experiments. Each log-logistic created convolution (A) was compared to the Skytrain and WAIS Divide CO_2 splines over a time window covering the entire CO_2 drop into the Little Ice Age by calculating the CO_2 offset of each at annual resolution. The Skytrain and WAIS divide splines have been adjusted by a consistent absolute CO_2 value to directly plot over the convolutions since the latter are based on the absolute values of Law Dome. The average offset is plotted (B) to indicate those functions which best fit the splines, where the offset is lowest.

Table S1: Summary information of each ice core CO_2 record and spline parameters. For spline average standard deviation calculations, the ends of the timeseries were cropped, as confidence intervals become artificially large here due to fewer data points.

	**Ice Core CO_2**					**Spline**				
	Timespan (yr CE)	Accumulation (cm water eq. yr)	Sample resolution (yrs) [1450-1650 CE]	Sample Error (ppm)	Age distribution (yrs)	Timespan (yr CE)	Data spacing (yrs)	Cut-off frequency (yrs)	Smoothing parameter	Av. Std deviation (ppm)
**Skytrain**	1688 - 1454	13.5	7.55 [7.69]	1.0	~ 27	1685 - 1455	1	50	2e-3	0.77
**Law Dome**	2006 - 13.3	60	7.97 [15.38]	1.1	8	1950 - 50	1	50	3e-3	1.14
**WAIS Divide**	1939 - 732	19.8	7.74 [6.66]	0.8	19	1900 - 750	1	50	1.3e-3	0.69
					**Compilation**	1950 - 50	1	100	8e-5	0.57

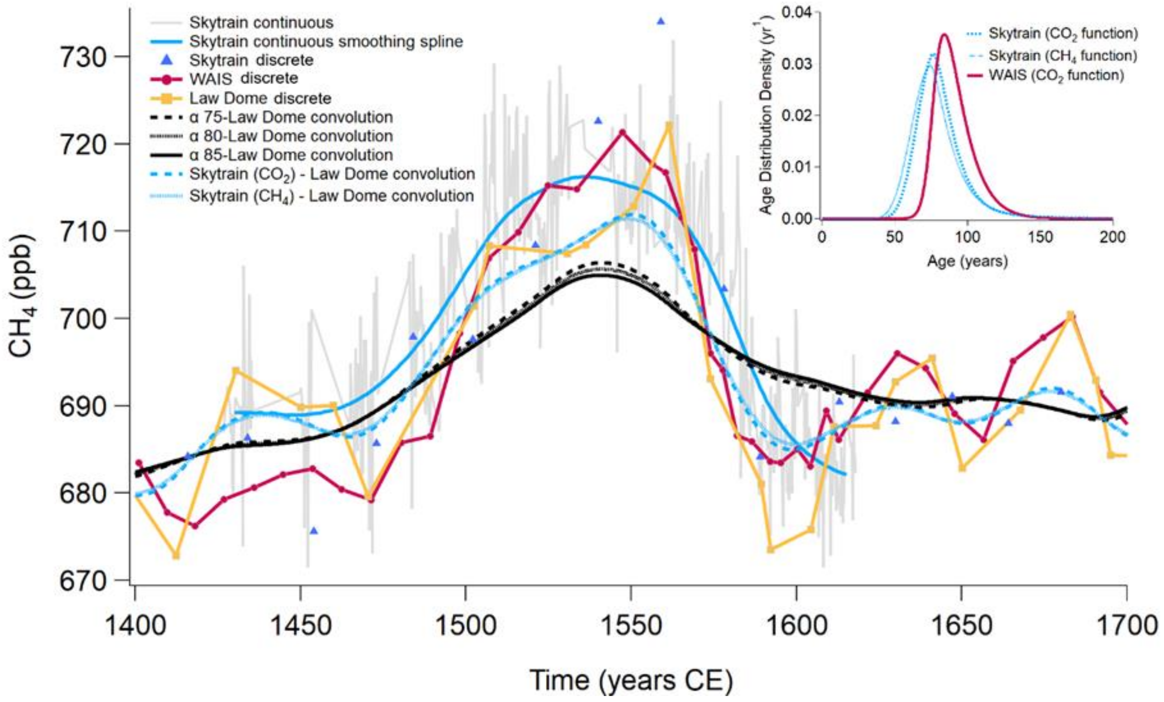

Figure S2: CH_4 convolution experiments showing effects of different diffusivity parameters on results. CH_4 convolutions as shown in Figure 3, main text, with a comparison convolution using a Skytrain firn filter generated using CH_4 diffusivity parameters rather than CO_2 diffusivity parameters (inset figure top right). The figure shows the effect of the changes in diffusivity parameter on both the firn filter output and the subsequent convolutions are negligeable. Also shown are the Skytrain continuous CH_4 at 30-second averages, and previous discrete CH_4 measurements [1].

Quantifying the mismatch between the data and the artificially smoothed CH_4 timeseries

To compare the CH_4 records we choose two representative windows: between 1480 and 1500 CE when CH_4 is increasing and 1560 and 1580 CE when CH_4 is decreasing. In the Skytrain ice core record, CH_4 first increases at 5.02 ppb/decade followed by a decrease of -6.39 ppb/decade in good agreement with the minimal, firn model-based smoothing (+4.61 ppb/decade and -6.18 ppb, respectively). In contrast, our three artificially enhanced smoothing filters clearly do not recreate the Skytrain record; decadal rates of change are greatly reduced. From 1480 to 1500 CE the rates are between 2.5 to 2.8 ppb/decade and between 1560 and 1580 CE range from -2.8 to -3.4 ppb/decade, around half of that of the rates observed in Skytrain record.

Late pre-Industrial atmospheric CO_2 compilation

We present a new synthesis of atmospheric CO_2 data from 1350 to 1800 CE. A previous compilation presented a spline record of CO_2 through this time-period as part of a complete 156 kyr smoothed CO_2 history [2] (Figure S3). The spline includes both the WAIS Divide and Law Dome records but adjusted the WAIS Divide data by -3.13 ppm to match the lower-offset Law Dome absolute values. With Skytrain absolute values sitting between both Law Dome and WAIS Divide, and with evidence that Law Dome values may be too low in specific areas, we instead adjust the Law Dome and WAIS Divide datasets to their average offsets to the Skytrain record, +1.36 ppm and -1.28 ppm respectively. For the Law Dome offset calculation, the three data points of the '1610 minimum' were removed so as not to introduce a low bias. When all three data sets are combined and smoothed with a 100-yr cut-off spline, the atmospheric CO_2 history shows a maximum CO_2 value of 284.4 ± 0.3 ppm at 1532 CE, declining to a minimum CO_2 value of 277.6 ± 0.2 ppm at 1673, with a decadal rate of decline in CO_2 of 0.5 ppm (Table 1). The preceding 'baseline' CO_2 is 281.5 ± 0.3 ppm at 1448 CE. The centennial scale CO_2 variability is highly comparable to the previous compilation, but the absolute values remain uncertain. Thus, we can be confident in the relative changes in climate model simulations forced by this compilation or previous compilations that excluded the '1610 minimum'.

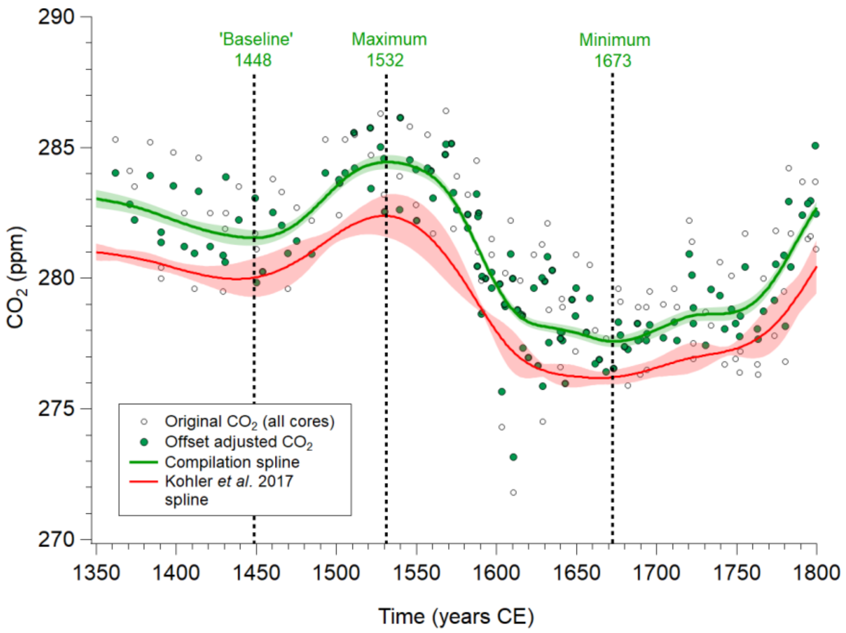

Figure S3: A compilation smoothing spline. Including the new Skytrain CO_2 record, shown comparative to a previously published spline using Law Dome and WAIS Divide only (red line) [2]. The spline (green line) is generated from a compilation of all three CO_2 datasets (original records, open circles), but with Law Dome and WAIS Divide values adjusted to account for their respective average offset to Skytrain (adjusted compilation records, green circles).

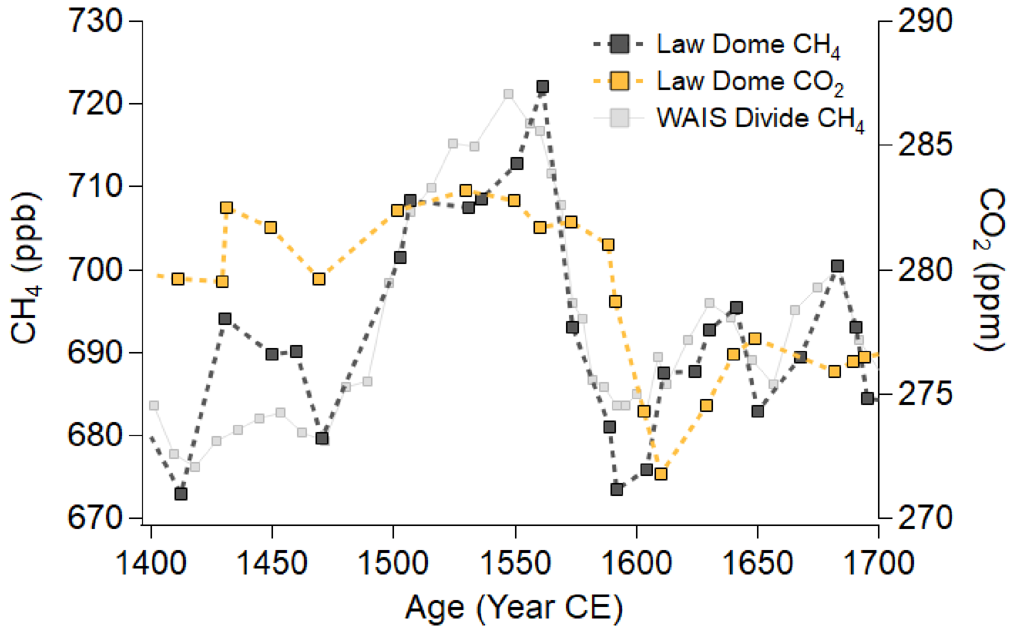

Figure S4: Comparison plot of CO_2 and CH_4 in Law Dome. Highlighting that a short, rapid drop in CH_4 is also present around 1600 CE, which is not present in the WAIS Divide (shown for reference) and Skytrain records. The duration of the CO_2 drop (~40 yrs) is about twice that of the duration of the CH_4 drop (~20 yrs).

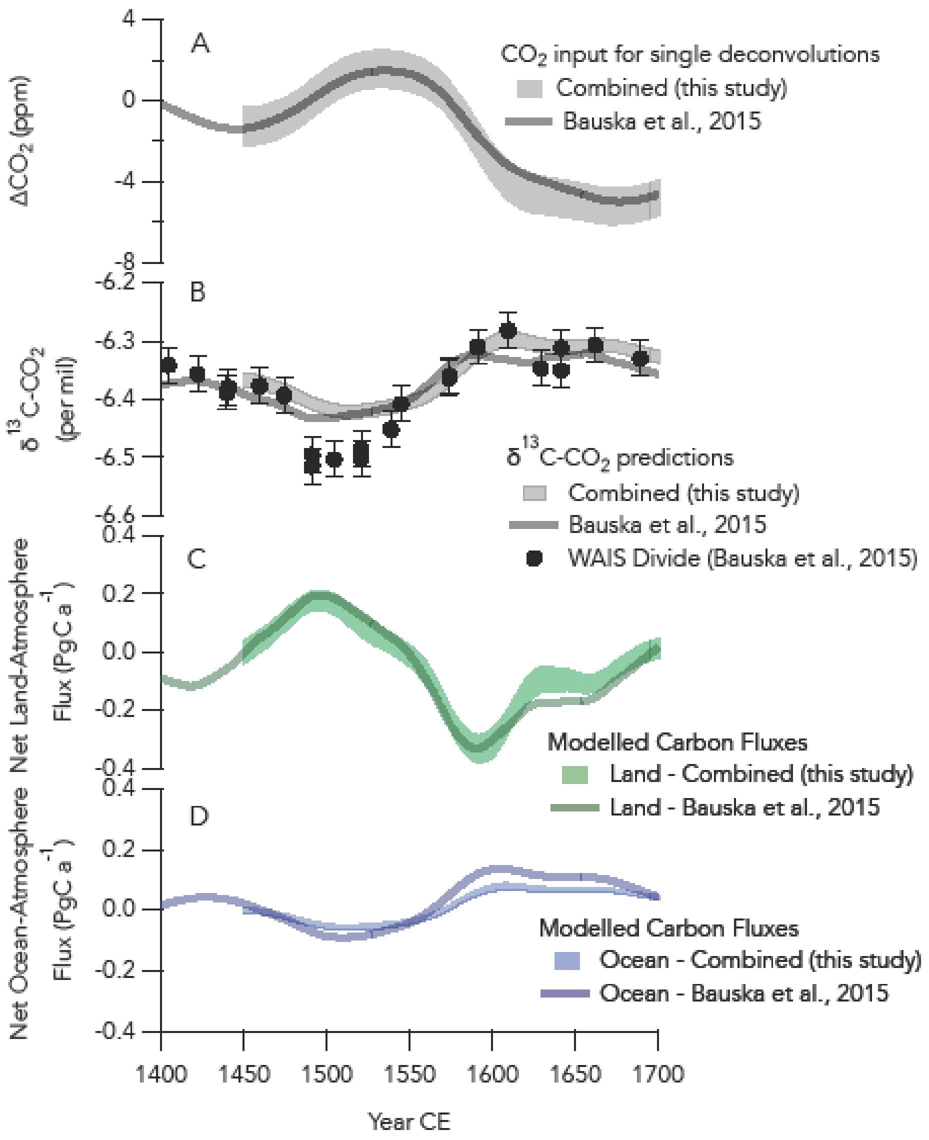

Figure S5: A comparison of the land and ocean carbon fluxes predicted in this study (shaded bands) with the OSU Carbon Cycle Model and a previous study using just the WAIS Divide ice core data and the HILDA model [3,4] (darker lines). All experiments are single deconvolution experiments and thus use only the atmospheric CO_2 data (A) to predict the land-atmospheric carbon flux (C). The atmospheric d13C-CO_2 with 1-σ s.d. error bars (B) and ocean-atmosphere fluxes (D) are allowed to freely evolve in the model. The single deconvolutions fail to capture the d13C-CO_2 minimum that precedes the 1610 drop but do capture the broader maximum within the top itself.

Supplementary References

1. Hoffmann, H. M. *et al.* The ST22 chronology for the Skytrain Ice Rise ice core - Part 1: A stratigraphic chronology of the last 2000 years. *Climate of the Past* **18**, 1831–1847 (2022).
2. Köhler, P. *et al*. A 156 kyr smoothed history of the atmospheric greenhouse gases CO2, CH4, and N2O and their radiative forcing. *Earth Syst Sci Data* **9**, (2017)
3. Joos, F., and Bruno, M. Long-term variability of the terrestrial and oceanic carbon sinks and the budgets of the carbon isotopes 13C and 14C, *Global Biogeochem Cycles* **12,** (1998).
4. Bauska, T. K. *et al.* Links between atmospheric carbon dioxide, the land carbon reservoir and climate over the past millennium. *Nat Geosci* **8**, (2015).
