# Supplementary material for: Reconciling ice core CO2 and land-use change following New World-Old World contact
Source: Nat Commun. 2024 Mar 5;15:1735. doi: 10.1038/s41467-024-45894-9 (PMC10915154; doi:10.1038/s41467-024-45894-9)
Supplement: Supplementary file 3 — Description of Additional Supplementary Files [file 41467_2024_45894_MOESM3_ESM.pdf]

Description of Additional Supplementary Files:

Supplementary Data 1: Measurements of CO_2 and CH_4 in the Skytrain ice core, gas record smoothed splines, and generated firn filters.

Supplementary Data 2: Outputs of the land carbon flux model.
