# Supplementary material for: Reconciling ice core CO2 and land-use change following New World-Old World contact
Source: Nat Commun. 2024 Mar 5;15:1735. doi: 10.1038/s41467-024-45894-9 (PMC10915154; doi:10.1038/s41467-024-45894-9)
Supplement: Supplementary file 2 — Peer Review File [file 41467_2024_45894_MOESM2_ESM.pdf]

nature portfolio

Peer Review File

Reconciling ice core CO2 and land-use change following New World-Old World contact

Open Access This file is licensed under a Creative Commons Attribution 4.0 International License, which permits use, sharing, adaptation, distribution and reproduction in any medium or format, as long as you give appropriate credit to the original author(s) and the source, provide a link to the Creative Commons license, and indicate if changes were made. In the cases where the authors are anonymous, such as is the case for the reports of anonymous peer reviewers, author attribution should be to 'Anonymous Referee' followed by a clear attribution to the source work. The images or other third party material in this file are included in the article's Creative Commons license, unless indicated otherwise in a credit line to the material. If material is not included in the article's Creative Commons license and your intended use is not permitted by statutory regulation or exceeds the permitted use, you will need to obtain permission directly from the copyright holder. To view a copy of this license, visit http://creativecommons.org/licenses/by/4.0/.

Reviewer #1 (Remarks to the Author):

This study uses a new ice-core CO2 record (Skytrain) and modelling to suggest that a previously reported brief dip in atmospheric CO2 seen around 1600 in the much-used Law Dome CO2 record is likely an artifact. Instead, although a drop did occur around that time, it was more gradual. This more gradual drop is more in line with some prior estimates of land CO2 fluxes that followed from (tragic) decimation of native American populations in around 1600 and resulting land abandonment.

I found the paper to be quite interesting, with implications for both anthropology and climate sciences, e.g. the 1600 dip has been previously highlighted as a marker for defining the Anthopocene (Lews and Manslin, 2015). I appreciated the (mostly) very clear writing and presentation. The argument is well crafted and generally convincing, especially because the new Skytrain core corroborates the CO2 record from the WAIS divide core, which I surmise was previously discounted because it disagreed with the favored Law Dome record. In effect, the Law Dome record is now outvoted. The authors skillfully address complications caused by smoothing of the different ice-core records by diffusion in the firn column. Overall, very nice work!

My only substantive concern relates to error analysis. Although the general agreement between the Skytrain and WIAS cores makes other details of error analysis less critical, I nevertheless feel the need to point out some issues. For one thing, the presentation wasn't super clear. But if I understood correctly, they apply a fixed +/- 1 ppm uncertainty to each data point, and propagate errors via a Monte Carlo scheme. The problem here is that the +/- 1 ppm estimate is by itself not well justified (see below) and may not fully capture relevant errors. I suggest that a better approach would be to use a resampling method, such as a bootstrap, using random picks of the data to feed into the spline fitting. There is no perfect method, but I believe a bootstrap will at least involve fewer arbitrary assumptions. I suggest that they do bootstrapping for all three ice cores.

Detailed comments:

184-185. I'm not sure this is correct. I believe the key requirement is for a regularly spaced atmospheric times series, whether smoothed or not. An interpolation method is thus needed, but it's not required that the resulting time series by highly smoothed.

Figure 4. I would appreciate seeing the time axis for these plots extending over a larger time frame, so that the dip around 1600 can be seen in the context of longer-term variability. Why not use the 1300-1800 timeframe shown in Figure 2?

236. The double negative and the phrase "high per-capita land use", which I found ambiguous on first reading, makes this hard to parse. I suggest rephrasing avoiding the double negative and using previously defined terms "high-end" and "low-end".

250-252. This needs some editing for clarity. As currently written, the 1600 cooling doesn't appear within the summary of cooling periods. I suggest simply ranking the cooling around 1600 in relation to other periods. I'm also not clear what is meant by coldest (non overlapping) decades.

288 The term "discrete CH4" unclear

309. NOAA revised its CO2 scale recently. What NOAA scale is being used?

316. See general point above about error analysis. It's unclear why pooled standard deviation is being used as an error metric. If errors were truly random, the pooled standard error (across replicates) would be more relevant, which would be sqrt(2) or sqrt(3) times more precise. As far as I can tell, using the +/- 1 ppm number for replicate averages has no real justification, or at least not one that has been disclosed. More to the point, it's clear from comparing the records in Figure 2 that other errors are dominating, such as systematic differences between the true atmospheric value and what's preserved in the ice or something to do with the extraction. A sensible error analysis would quantify these aspects rather than reporting a statistic related to

random error on a single point that is then applied to a replicate average. As I suggested earlier, a bootstrap would be more inclusive of different types of error with fewer assumptions.

347. What's a compilation spline?

348. See earlier point. I don't follow this method. Was a constant with sigma +/- 1 ppm added for each sample point? Based on my earlier comment, I doubt these addresses relevant errors.

361. Not clear what is meant by "conservative value". High or low?

368. What is y in this equation?

Reviewer #3 (Remarks to the Author):

Review of "Reconciling ice core CO_2 and land-use change following New World-Old World contact" King, A.C.F. et al., submitted to Nature Communications.

This manuscript by Amy King et al. assesses atmospheric CO_2 changes measured in ice cores during the pre-industrial centuries. This is an important period as it shows variations in the carbon cycle before large emissions from fossil fuel that may be evidence of natural, likely climate-driven, causes or early anthropogenic land use change. It has been the focus of several ice core measurement surveys and modelling interpretation studies, in particular the CO_2 minimum at about 1590-1630 AD. King et al. present another ice core record through this period from the relatively new Skytrain ice core. They question whether the CO_2 minimum observed in the Law Dome DSS ice core is an artefact as opposed to an actual atmospheric change.
As the authors note, variations in CO_2 at this time have been widely used in quantifying climate, carbon cycle and anthropogenic roles and as such we need to be sure of their accuracy.
The main requirements to produce a CO_2 record from ice cores that best represents rapid atmospheric variations are that the measurements are an accurate measure of the CO_2 concentrations of the air in the ice, that the CO_2 in the ice has not changed since enclosure in bubbles and that the enclosure of air in the bubbles has minimal effect on the CO_2 concentration of the atmosphere of the time.
The measurements by King et al. appear to be done well and have replicates and high measurement density through this period. However, the resulting scatter of CO_2 concentrations appears to be rather large compared to their measurement precision, the likely atmospheric variability and, importantly, the likely variability that would remain after the smoothing effect of air enclosure in the ice at the Skytrain site. This is discussed further next.
Variations in the Skytrain CO_2 measurements are sometimes quite large and appear difficult to reconcile as being atmospheric in origin given our knowledge of likely variations in atmospheric CO_2 and additionally the attenuation of rapid atmospheric signals by the ice core air enclosure process. For example, point-to-point differences (separated by less than a decade) of 4-5 ppm occur at around 1540 and (importantly) 1610-1630 AD. Almost as much CO_2 variability is seen in WAIS in the early 1600s. Is this evidence that these cores are affected in some way? This variability is comparable to the difference between the measured CO_2 minimums in the Skytrain and WAIS cores and the expected CO_2 minimum if the Law Dome record were convolved by their respective air age distributions (blue and red data and lines in Figure 3). Note also that both Skytrain and WAIS are slightly elevated in CO_2 compared to many other ice core records: Law Dome, DML, EPICA/DML (Rubino et al., ESSD, 2019) and South Pole (Siegenthaler et al., 2005). Is this revealing anything important about CO_2 retention in WAIS or Skytrain? The WAIS-Law Dome difference was found not to be caused by inter-laboratory measurement differences (Ahn et al., GBC, 2012). So while it is reasonable to question some of the lower values within the DSS record, a similar level of scrutiny could also be applied to the Skytrain CO_2 measurements, especially if they align with the WAIS record that is likely elevated.
A key question is how an ice core with an accumulation of only 13.5 cm/yr can retain atmospheric composition variations that can be used to verify a rapid variation observed in the higher accumulation rate ice from Law Dome (DSS, 60 cm/yr)? The spread of air age in ice is a result of the diffusion of air in the firn column and additional smoothing as the air deep in the firn is

enclosed into bubbles. That additional smoothing can be significant and would be larger for low accumulation sites. It is not clear from the manuscript how this additional smoothing has been included for Skytrain, if at all, in the resulting air age distributions used in the analysis and comparison of potential CO2 changes observed in these different ice cores. There are many references in the text to the advantages of high accumulation and temporal resolution (for example, paragraph staring at line 38) and then testing of Law Dome CO2 through the firn based smoothing (section at line 114). But I couldn't find what was included in the "firn-filters" (line 129). Firn-filter modelling is described at line 353, and mentions densification and close-off, but does it include bubble close-off, either measurement or parameterisation, and its effect on air age spread?
Validation of ice core air age distributions is useful to quantify the effect of enclosure smoothing on atmospheric variations. One approach is to test the air age distributions derived for the air in ice core bubbles against rapid atmospheric changes of gas species with independently found histories. For example, 14CO2 measurements of Law Dome ice core and firn air, including the DSS ice core, were compared with measurements of the "bomb-pulse" 14CO2 in the atmosphere smoothed by the model of firn diffusion and bubble close-off (Smith et al., NIMB, 2000). Have measurements of any gas species other than CO2 or CH4 been used to check the Skytrain ice core air age distributions?
King et al. test whether the smoothing required to remove all of the 1610 CO2 minimum signal is compatible with the CH4 measurements (from line 149). This makes good sense as there are CH4 data through these periods for the 3 cores and CH4 is generally reliably enclosed and stored in air in ice. However, this comparison depends on how well constrained are the WAIS and Skytrain age distributions ("firn filters"). If they are trained on matching the CO2 datasets would they be potentially in error due to the variability that both cores' CO2 measurements show especially in the 1600s? It's also difficult to see how well the increased smoothing functions fit the data (Fig 3B). These are quite radical increases in smoothing and it would help to see the CO2 data and their uncertainties plotted alongside. The CH4 data could also be shown in Fig 3C as well as the spline. It should be noted that there are rapid CH4 variations in Law Dome DSS that are not observed in either Skytrain or WAIS, curiously also around 1600AD. It would be unlikely that these CH4 differences were caused by the same artefact that is proposed to have affected CO2.
The general approach of King et al. is similar in many ways to that used by Rubino et al., Nature Geo 2016, who compared the CO2 changes in the WAIS and DML (also lower accumulation than Law Dome) ice cores with those in the Law Dome DSS core. The CO2 measurements were consistent with the DSS CO2 record convolved through the bubble air age distributions for both those ice cores but neither could confirm or rule out the 1610 minimum in DSS. Without evidence that the Skytrain bubble air age distribution is sufficiently narrow and not broadened by its relatively low accumulation rate, it is difficult to see how it could offer a significant improvement.
A final general comment regards the comparison of the Law Dome 1610 minimum with carbon fluxes from land use change scenarios. The authors interpret the inability of land use models to account for the measured 1610 minimum as evidence that it is implausibly large. It is interesting to make model-data comparisons like these but I think the land use estimates for that time could also be uncertain. More importantly, this claim seems to ignore the CO2, 13CO2 and COS measurements and modelling presented by Rubino et al. (Nature Geo., 2016) as evidence for a climate role in the Little Ice Age CO2 drop (~1600-1800).
Arguing that a low accumulation ice core can better record rapid atmospheric CO2 variations than a higher accumulation counterpart requires convincing demonstration that the air age distribution has been fully accounted for and that there are no artefacts in the ice core measurements- of both high and low accumulation cores. King et al. go some of the way in doing this for their Skytrain study but more is required to make their claims more robust. Given the importance of ensuring that past CO2 variations are accurately reconstructed from ice cores I hope that they can make the suggested improvements. Their final statement that new very high accumulation (air age resolution) Antarctic ice cores are needed must stand as a high priority.

Other, specific notes on the manuscript:
Lines 45, 46, 53, 55. Please say whether and how bubble close off is accounted for in these estimates, and later on in the analysis.
Line 56. 0-6 ppm is given in Ahn et al.
Line 60. Is this saying that both records could have artefacts?
Line 68, 77. Should also say that the other interpretation is climate-CO2 feedback.

Line 80. Prefer “interpretation” or “understanding” to accounting.
Line 94. More information on Skytrain relevant to ice core CO2 would be helpful: temperature, melt events, age and uncertainty, locations, how drilled…. Either here or in Methods/Supplementary.
Line 100. The first part of the CO2 decrease actually looks comparable to Law Dome.
Line 109. As in general comments above, regions of large variability in both Skytrain and WAIS.
Line 117. Low pass filtering occurs in firn and bubble close of region.
Line 119. Are all air age distributions using the same statistic?
Line 122. For clarification: …(about 40 years) was ana actually atmospheric CO2 change recorded in Law Dome ice but smoothed away in both Skytrain and WAIS?...
Line 128. “Firn filters” are also referred to as Greens Functions for the purpose of convolutions and forward and inverse modelling signals.
Line 131, 132. The convolved minimums are much more subtle than the originals, and are actually not much different from the data, especially Skytrain, given the stated uncertainties and the additional variability.
Line 138. Again, what is included in the firn filters?
Line 150. If it is a true signal and if it is enclosed and preserved without unexpected changes…
Line 158. Could the Skytrain CH4 data be plotted please?
Figure 3B. Could the CO2 data be plotted to see how well the convolutions fit?
Figure 3C. Please show Skytrain data points.
Figure 4. It would be help if the ocean-atmosphere fluxes were shown or at least mentioned.
Line 161. Given the points raised above this would be better stated as “a plausible”.
Line 232. Also as stated above, there are other possible reasons for the CO2 change.
Line 261, 262. Based on the reasons given above, this statement is possibly too confident. Suggest removing the word “strong” unless more evidence as mentioned earlier can be shown.
Line 292. What is the range/uncertainty of the CH4 blank?
Section at line 355. Please be clear about what processes are included in the firn filter modelling, especially bubble close off and how it interacts with the diffusion in the firn column. This is crucial to the resulting air age spread and the conclusions of this work.

REVIEWER COMMENTS

Author responses are given in blue text.

Reviewer #1 (Remarks to the Author):

This study uses a new ice-core CO2 record (Skytrain) and modelling to suggest that a previously reported brief dip in atmospheric CO2 seen around 1600 in the much-used Law Dome CO2 record is likely an artifact. Instead, although a drop did occur around that time, it was more gradual. This more gradual drop is more in line with some prior estimates of land CO2 fluxes that followed from (tragic) decimation of native American populations in around 1600 and resulting land abandonment.

I found the paper to be quite interesting, with implications for both anthropology and climate sciences, e.g. the 1600 dip has been previously highlighted as a marker for defining the Anthopocene (Lews and Manslin, 2015). I appreciated the (mostly) very clear writing and presentation. The argument is well crafted and generally convincing, especially because the new Skytrain core corroborates the CO2 record from the WAIS divide core, which I surmise was previously discounted because it disagreed with the favored Law Dome record. In effect, the Law Dome record is now outvoted. The authors skillfully address complications caused by smoothing of the different ice-core records by diffusion in the firn column. Overall, very nice work!

Thank you very much to the reviewer for the positive feedback, and for the constructive comments given below.

My only substantive concern relates to error analysis. Although the general agreement between the Skytrain and WIAS cores makes other details of error analysis less critical, I nevertheless feel the need to point out some issues. For one thing, the presentation wasn't super clear. But if I understood correctly, they apply a fixed +/- 1 ppm uncertainty to each data point, and propagate errors via a Monte Carlo scheme. The problem here is that the +/- 1 ppm estimate is by itself not well justified (see below) and may not fully capture relevant errors. I suggest that a better approach would be to use a resampling method, such as a bootstrap, using random picks of the data to feed into the spline fitting. There is no perfect method, but I believe a bootstrap will at least involve fewer arbitrary assumptions. I suggest that they do bootstrapping for all three ice cores.

We have updated our analysis to include bootstrap for producing our smoothing splines of all ice core records and the confidence intervals (error) on these splines.

We use a 'true' bootstrap method, random sampling with replacement, and include the analytical uncertainties in the bootstrap. We chose this over random sampling without replacement as this method would be overly sensitive for shorter time series, which our Skytrain data set is. The generated splines and their confidence intervals now replace all previous splines used in the figures, smoothing experiments, and land carbon modelling in our manuscript, but do not change any of the conclusions of our work. To demonstrate this, Figure R1A shows a comparison between our previous splines and their confidence intervals and the new bootstrap method splines and their confidence intervals. The figure shows the increased capture of data variability in the confidence intervals as requested by the reviewer, while overall splines remain virtually the same. Figure R1B highlights the similarity of the splines in our period of interest, the same as they are used in manuscript Figure 2 for our convolution experiments. These new splines do not impact the conclusions of any of our smoothing experiments. Finally, Figure R1C shows the new land carbon modelling outputs, based on the bootstrap splines and confidence intervals, alongside the previous outputs. There are subtle

differences in the confidence intervals of calculated land - atmosphere fluxes due to the now wider input confidence intervals of our splines, but overall trends in the fluxes are not changed.

A note that the confidence intervals on our new bootstrap splines may become very wide at the end of the datasets as shown in Figure R1A for Skytrain since this time series is shorter. The ends of the time series are constrained by fewer data points resulting in wider errors here. This does not affect the data or splines/confidence intervals in our specific period of interest in the core. The same end-effects occur for all records but lie outside the window of this particular plot, and are reduced in the case of WAIS Divide and Law Dome given they are longer datasets overall.

The methods section which reports the generation of our smoothing splines has been updated to reflect the new bootstrap methodology used.

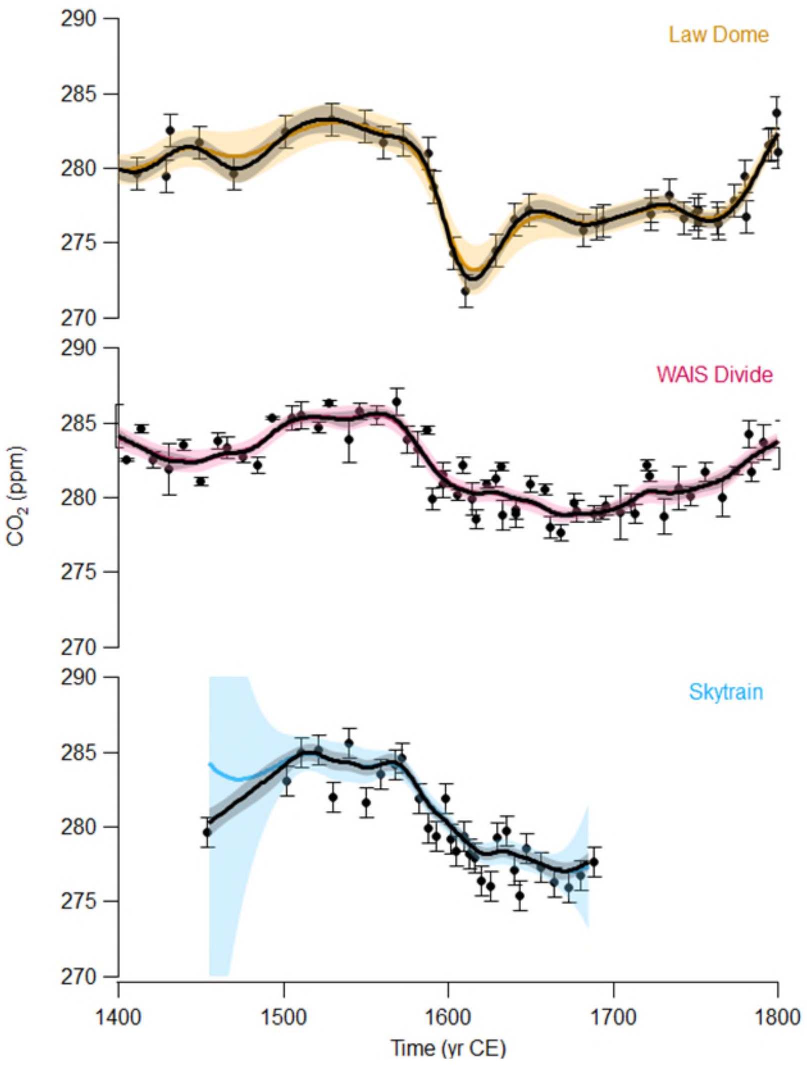

Figure R1A: CO_2 data for each of the three cores in this study, with their previous monte carlo smoothing splines and confidence intervals shown in black/grey bands, and the new bootstrapped smoothing splines and confidence intervals in coloured bands. Confidence intervals become very wide at the end of the datasets as shown for Skytrain here since this time series is shorter. The ends of the time series are constrained by fewer data points resulting in wider errors. The same end-effects occur for all records but lie outside the window of this particular plot, and are reduced in the case of WAIS Divide and Law Dome given they are longer datasets overall.

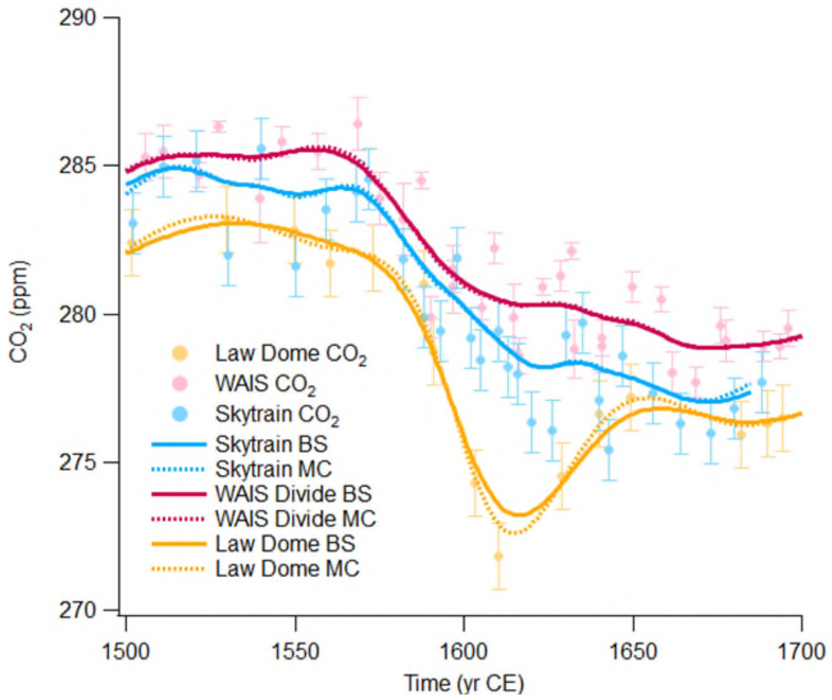

Figure R1B: CO_2 data for each of the three cores through the period of interest to this study, in the style of manuscript Figure 2 for comparison. The graph plots the previously used splines generated using the monte carlo (MC) method (dashed lines), with the new splines generated using the bootstrapped (BS) method (solid lines). There is a strong similarity between both versions of the splines, thus the conclusions of our later smoothing experiments are not affected.

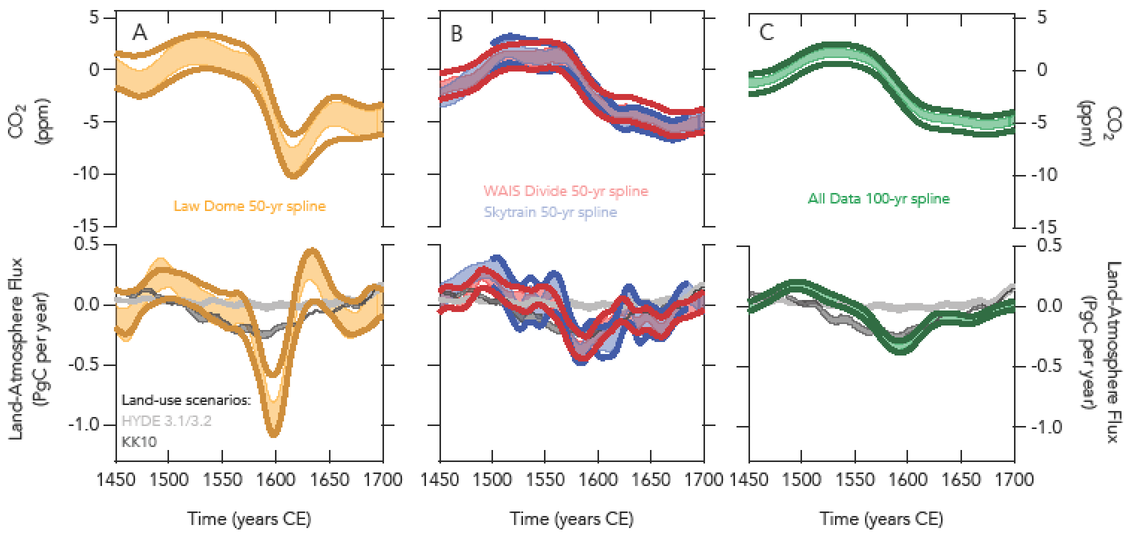

Figure R1C: A comparison of results from the deconvolution experiments for the various ice core records showing the change in CO_2 and the inferred land-atmosphere carbon flux based on our previous splines and confidence intervals (coloured shading) and fluxes based on our updated bootstrapped splines and confidence intervals (bold lines). There are some subtle differences in the confidence intervals of the calculated fluxes due to the new confidence intervals of our splines being generally of increased width, however overall trends in fluxes remain the same.

Detailed comments:

184-185. I'm not sure this is correct. I believe the key requirement is for a regularly spaced atmospheric times series, whether smoothed or not. An interpolation method is thus needed, but it's not required that the resulting time series by highly smoothed.

Good point. We have modified the sentence to read *'This technique requires a continuous record of atmospheric CO_2, interpolated to the model timestep, which we take from the suite of bootstrapped spline fits.'*

Figure 4. I would appreciate seeing the time axis for these plots extending over a larger time frame, so that the dip around 1600 can be seen in the context of longer-term variability. Why not use the 1300-1800 timeframe shown in Figure 2?

Thank you for the suggestion. We have retained the original time axis range as this covers the full extent of out new Skytrain record. A fuller Common Era picture is outside the scope of our dataset and this study.

236. The double negative and the phrase "high per-capita land use", which I found ambiguous on first reading, makes this hard to parse. I suggest rephrasing avoiding the double negative and using previously defined terms "high-end" and "low-end".

Thanks for pointing out the confusion. We have reworded as follows *'This challenges previous work which, based on the longer-term carbon balance over the last millennium [4], suggested the "high-end" scenarios were implausible, as well as recent work which revised downward the per-capita land use in the Americas [11].'*

250-252. This needs some editing for clarity. As currently written, the 1600 cooling doesn't appear within the summary of cooling periods. I suggest simply ranking the cooling around 1600 in relation to other periods. I'm also not clear what is meant by coldest (non overlapping) decades.

We have added further description to clarify:

'Intriguingly, many tree-ring based reconstructions of Northern Hemisphere temperature record a strong cooling around 1600 CE, but this oscillation is not particularly exceptional in the wider context of decadal-scale cold periods of the last millennium. For context, the two coldest decades of the last millennium are in the 1800s followed by 1462-1471, 1695-1704 and 1452-1461 as the 3rd, 4th and 5th coldest, respectively[31]. Thus, if the terrestrial carbon cycle was particularly sensitive to rapid coolings, most crucially, with a response time capable of driving a decadal-scale drop in CO_2 as observed in the Law Dome ice core, we would expect similarly large CO_2 decreases following 1450/60s and the early 1700s. Instead, CO_2 is either stable or even increasing during these intervals. More likely, a series of coolings starting as early as the 1300's in the Northern Hemisphere (in particular the Arctic) contributed to a gradual uptake of carbon on land and no exceptional carbon cycle feedback is required around 1610 CE.'

288 The term "discrete CH4" unclear

Changed 'discrete CH_4' to *'Discrete (non continuous) samples…measured for CH_4'.*

309. NOAA revised its CO2 scale recently. What NOAA scale is being used?

Added to sentence: *'(WMO scale X2019)'.*

316. See general point above about error analysis. It's unclear why pooled standard deviation is being used as an error metric. If errors were truly random, the pooled standard error (across replicates) would be more relevant, which would be sqrt(2) or sqrt(3) times more precise. As far as I can tell, using the +/- 1 ppm number for replicate averages has no real justification, or at least not one that has been disclosed. More to the point, it's clear from comparing the records in Figure 2 that other errors are dominating, such as systematic differences between the true atmospheric value and what's preserved in the ice or something to do with the extraction. A sensible error analysis would quantify these aspects rather than reporting a statistic related to random error on a single point that is then applied to a replicate average. As I suggested earlier, a bootstrap would be more inclusive of different types of error with fewer assumptions.

We now use a bootstrap method for generation of splines and confidence intervals as suggested (please see more detailed response to error analysis above). The pooled standard deviation remains to indicate analytical error bars on our individual CO_2 data points. The pooled standard deviation was chosen as it is widely used in reporting of error on ice core gas measurements, and because of our variation in replicate numbers across data points.

347. What's a compilation spline?

Changed to *'...the spline based on a compilation of the datasets.'*

348. See earlier point. I don't follow this method. Was a constant with sigma +/- 1 ppm added for each sample point? Based on my earlier comment, I doubt these addresses relevant errors.

As in previous comments, this method has been updated to include bootstrap and appropriately address errors.

361. Not clear what is meant by "conservative value". High or low?

'Conservative' has been removed to avoid introducing confusion.

368. What is y in this equation?

y is the term used to represent the log logistic distribution here. To clarify this we have added in brackets in line 366: *'Log logistic distributions (y) can also be....'*

Reviewer #3 (Remarks to the Author):

This manuscript by Amy King et al. assesses atmospheric CO2 changes measured in ice cores during the pre-industrial centuries. This is an important period as it shows variations in the carbon cycle before large emissions from fossil fuel that may be evidence of natural, likely climate-driven, causes or early anthropogenic land use change. It has been the focus of several ice core measurement surveys and modelling interpretation studies, in particular the CO2 minimum at about 1590-1630 AD. King et al. present another ice core record through this period from the relatively new Skytrain ice core. They question whether the CO2 minimum observed in the Law Dome DSS ice core is an artefact as opposed to an actual atmospheric change.

As the authors note, variations in CO2 at this time have been widely used in quantifying climate, carbon cycle and anthropogenic roles and as such we need to be sure of their accuracy.

The main requirements to produce a CO2 record from ice cores that best represents rapid atmospheric variations are that the measurements are an accurate measure of the CO2

concentrations of the air in the ice, that the CO2 in the ice has not changed since enclosure in bubbles and that the enclosure of air in the bubbles has minimal effect on the CO2 concentration of the atmosphere of the time.

Thank you to the reviewer for their insightful comments on our manuscript and further discussion of the points raised. We have improved the manuscript as suggested, with the details of these changes found below.

The measurements by King et al. appear to be done well and have replicates and high measurement density through this period. However, the resulting scatter of CO2 concentrations appears to be rather large compared to their measurement precision, the likely atmospheric variability and, importantly, the likely variability that would remain after the smoothing effect of air enclosure in the ice at the Skytrain site. This is discussed further next.

Variations in the Skytrain CO2 measurements are sometimes quite large and appear difficult to reconcile as being atmospheric in origin given our knowledge of likely variations in atmospheric CO2 and additionally the attenuation of rapid atmospheric signals by the ice core air enclosure process. For example, point-to-point differences (separated by less than a decade) of 4-5 ppm occur at around 1540 and (importantly) 1610-1630 AD. Almost as much CO2 variability is seen in WAIS in the early 1600s. Is this evidence that these cores are affected in some way? This variability is comparable to the difference between the measured CO2 minimums in the Skytrain and WAIS cores and the expected CO2 minimum if the Law Dome record were convolved by their respective air age distributions (blue and red data and lines in Figure 3). Note also that both Skytrain and WAIS are slightly elevated in CO2 compared to many other ice core records: Law Dome, DML, EPICA/DML (Rubino et al., ESSD, 2019) and South Pole (Siegenthaler et al., 2005). Is this revealing anything important about CO2 retention in WAIS or Skytrain? The WAIS-Law Dome difference was found not to be caused by inter-laboratory measurement differences (Ahn et al., GBC, 2012). So while it is reasonable to question some of the lower values within the DSS record, a similar level of scrutiny could also be applied to the Skytrain CO2 measurements, especially if they align with the WAIS record that is likely elevated.

Figure R3A, below, shows a running three-point standard deviation of the CO_2 data from each of our three cores alongside the CO_2 records of the cores as originally shown in manuscript Figure 1. The standard deviations show that all three cores display periods of higher and lower variability (or, point-to-point differences) with comparable overall ranges of this variability. Throughout the '1610' period in particular, variability of Skytrain is not outside the range of the other two cores. Highlighted in the boxes overlying the CO_2 records are periods where each core record shows high variability even though the suggested background atmospheric CO_2 record is relatively unchanging. Data variability above that of the analytical precision, and presumably not of atmospheric origin, is something that persists across ice core records and is not fully explained. However, given that each of the three cores used in this study show good analytical precision and evidence of overall faithful capturing of atmospheric trends, they all maintain as excellent atmospheric records. It is difficult to analyse variability between the cores any further than this due to differences in sample resolution and analytical methods applied.

We certainly agree with the reviewer that a similar level of scrutiny can be applied to variability in each of the cores, but we hope this demonstrates that the Skytrain record is of comparably good quality to both Law Dome and WAIS Divide, in particular through the '1610' time period. To more markedly capture the observed variability in the cores through our error analysis, as suggested by Reviewer 1, we now include bootstrapping in our spline procedure for confidence intervals. Thus, we

show a fair reporting of the variability seen in each of the cores. Please see the description in response to Reviewer 1 for this, and Figure R1A.

As the reviewer points out, the offset between absolute values of the WAIS Divide and Law Dome records could not be connected to analytical differences, and no conclusion on the reason behind this has been drawn with certainty. The new Skytrain record does appear to be closer to WAIS Divide values preceding the CO_2 drop through the 1600s, but throughout the drop is not elevated alongside WAIS Divide and agrees with Law Dome in the short period measured following the CO_2 drop (also evidenced in Figure 1 showing the offset between WAIS Divide and Skytrain). Extension of the Skytrain CO_2 record outside of this time window was beyond the scope of this study and therefore it is not possible to call where the absolute values of Skytrain sit in the wider picture of the Common Era. It is thus difficult to further the discussion on the absolute offsets. However, in the rest of our study we present robust analyses to demonstrate that both WAIS Divide and Skytrain are faithfully recording the trends in atmospheric CO_2 at the time. Further, with the weighting of both the WAIS Divide core and the Skytrain core, which are from different locations, different coring, transport and storage conditions, and different analytical conditions, now in favour of a more reduced drop in CO_2 into the Little Ice Age despite their different absolute offsets, we demonstrate that absolute values are unlikely to be impacting our conclusions.

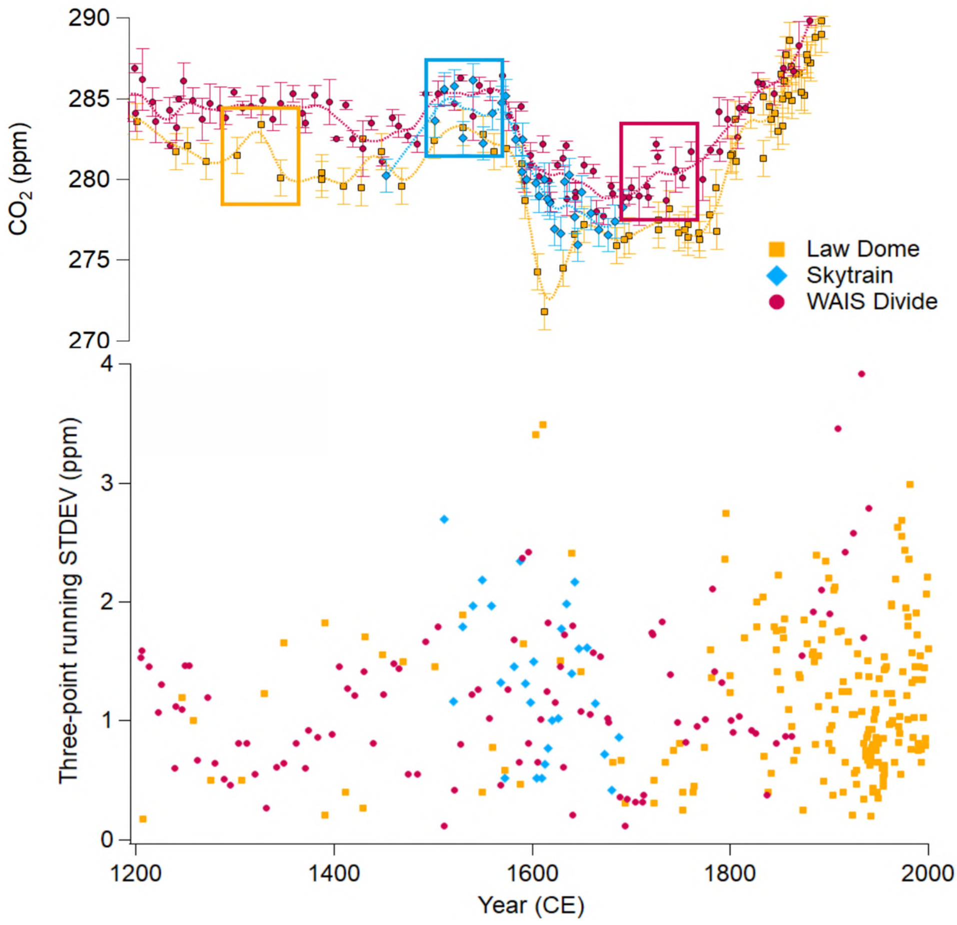

Figure R3A: Figure showing the CO_2 records (top) of Law Dome, WAIS Divide and Skytrain and (bottom) the corresponding running three-point standard deviation of each record, to capture point-to-point variability of the data. Overlying boxes highlight periods of high variability in sections of each of the three cores where background CO_2 trends otherwise appear relatively stable.

A key question is how an ice core with an accumulation of only 13.5 cm/yr can retain atmospheric composition variations that can be used to verify a rapid variation observed in the higher accumulation rate ice from Law Dome (DSS, 60 cm/yr)? The spread of air age in ice is a result of the diffusion of air in the firn column and additional smoothing as the air deep in the firn is enclosed into bubbles. That additional smoothing can be significant and would be larger for low accumulation sites. It is not clear from the manuscript how this additional smoothing has been included for Skytrain, if at all, in the resulting air age distributions used in the analysis and comparison of potential CO2 changes observed in these different ice cores. There are many references in the text to the advantages of high accumulation and temporal resolution (for example, paragraph staring at line 38) and then testing of Law Dome CO2 through the firn based smoothing (section at line 114). But I couldn't find what was included in the "firn-filters" (line 129). Firn-filter modelling is described at line 353, and mentions densification and close-off, but does it include bubble close-off, either measurement or parameterisation, and its effect on air age spread?

To address these concerns, we have improved our description of the firn air modelling procedure, better emphasised our existing tests which show the accuracy of our firn filters, and implemented additional description in the manuscript constraining the accuracy further.

As a first step, we acknowledge that in the main text it was not explicitly stated how the firn filters were generated when they are first mentioned, nor was it explicitly linked to the methods section which describes this, and therefore we have added: *'These firn filters, or age distributions, were generated using the Oregon State University (OSU) firn model (see methods) and mimic the firn smoothing specific to each ice core site.'*

As a second step, we discuss here some further detail on the firn filter methodology, and include additional information in the manuscript to make the generation of our firn filters clearer. The Oregon State University (OSU) Firn Model is a comprehensive firn model but, as in all firn models, the precise parameterisations will vary in comparison to other firn models. The performance of the OSU firn model in accurately reproducing firn smoothing on gas records was validated by testing reproducibility of firn air measurements in a Greenland borehole as reported in Buizert et al. 2012. This work also provided an intercomparison of multiple firn air models, including the OSU firn model, the CIC firn model (which produced the published WAIS Divide firn filter in Mitchell et al. 2015, to which we compare our OSU firn filter for our own reproducibility test as described in our manuscript), and the CSIRO firn model which was used to produce the Law Dome firn filter of Rubino et al. 2016. The intercomparison study of Buizert et al. 2012 shows good reproducibility between all models, and details their individual parameterisations.

For bubble closure specifically, the OSU firn model uses the bubble closure parameterisation of Buizert 2011, and as further detailed in Rosen et al. 2014, "concentrations are calculated assuming that closed bubbles pressurize at the same rate as bulk firn compaction". The OSU model therefore does not include more complex processes such as layering or other mechanisms which would result in variation in close-off depth. However, Mitchell et al. 2015 undertake an extensive investigation into these more complex processes, which are included in their (CIC) WAIS divide firn filter, and to which our (OSU) WAIS divide firn filter output shows very close reproducibility (Figure 2B). Thus, differences in bubble closure processes in the firn models do not appear to affect our results.

In the manuscript methods we have further added: *'The model incorporates physical processes of diffusion, advection, mixing, bubble closure and bubble compaction. The model has been validated using reproduction of Greenland firn air measurements and intercomparison with multiple other firn models shows good agreement in their depiction of smoothing under the same conditions [41]. For*

specific details on parameterisations please see references [41-43].' This is to give an overview of the included parameters and to reference the existing literature more clearly on the model.

Where we describe the WAIS Divide firn filter comparison in the methods, we also add: *'The CIC firn filter accounts for more complex processes of bubble closure, for example due to layering, in comparison to the OSU model which more simply defines bubble closure following the rate of bulk firn compaction [42]. Despite these differences the two firn filters very closely agree (Figure 2b), helping to validate our firn filters and indicating that differences in parameterisations between firn models are not impacting our results.'*

We have also highlighted the different bubble closure parameterisation of the comparison CIC WAIS Divide firn filter in the main manuscript where we present the firn filter comparison in Figure 2, adding to the caption *'alongside a previously published WAIS Divide filter that includes a stochastic model of bubble closure to mimic layering to show reproducibility'.*

We accept an error is present in all firn models due to an imperfect knowledge of firn processes. We have already built this assumption into our work with experiments of artificially enhanced smoothing. To state this potential error more explicitly at the first reporting of our smoothing experiments in the manuscript we have added *'could our firn filters be underestimating the degree of smoothing in the cores?'*. To connect these potential errors to specific model parameterisations, we further add *'For example, our firn model does not account for small-scale (cm-scale) density variations from layering and associated effects on bubble closure which can drive some pores to close-off about the traditional lock-in zone and thus lead to age-reversals and slightly broader firn age distributions.'*

Our tests of artificially enhanced smoothing account for error in the firn models which would result in an underestimation of the amount of smoothing of our records, or, place an upper limit on the firn filter widths. The results of these tests show that significantly increased smoothing, or width of our firn filters, is not compatible with the gas records we have measured. To further improve the constraints on the accuracy of our firn filters, we now also add to the manuscript a discussion on constraining the lower limit of the firn filter widths, adding confidence that out filters are within an accurate window:

'In an alternative approach, one could use the CH_4 records from all three cores to determine age distributions of WAIS Divide and Skytrain independently of the CO_2 record and provide not only estimates of the upper limits of the firn age widths (as done here), but also the lower limits. However, this would require high-resolution, continuous CH_4 data from Law Dome. Tentatively, we can place some constraints on the lower limit of the firn age distributions by examining a CH4 minimum that immediately precedes the CO_2 drop (~1590 CE). This minimum is fortuitously nearly coincident with the CO_2 drop of interest (Figure S4 (or R3B here), about half the width of the CO_2 drop (~20 years versus ~40 years), and about the same width as the gas age distribution of Skytrain and WAIS Divide. If our original estimates of smoothing based on firn models were accurate, we would predict that CH_4 minimum would be absent in WAIS Divide and Skytrain. This is clearly the case in the WAIS Divide record and most likely the case in the Skytrain record (although our measurement unfortunately did not extend to the predicted levelling off in CH_4 post 1600 CE (Figure 3). However, other features present in the low-resolution Law Dome data are not obviously comparable to the WAIS Divide and Skytrain features such as divergence in the preceding CH_4 bump of the 1500s CE. Measurements of CH_4 and other gases at the Law Dome site via continuous flow analysis (CH_4 CFA publications) are thus a crucial prerequisite for future work on understanding the smoothing imparted on ice core gas records by firn processes.'

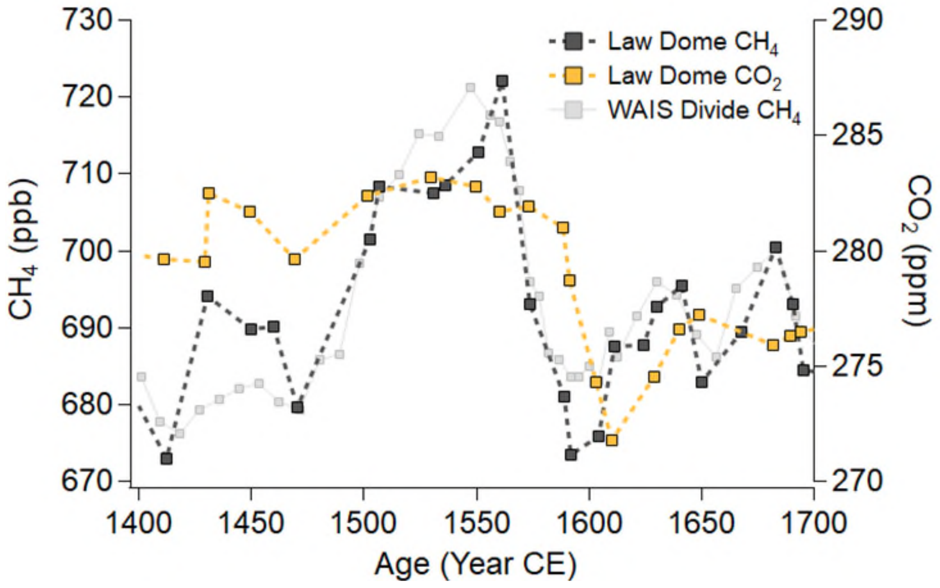

Figure R3B: Comparison plot of CO_2 and CH_4 in Law Dome to highlight that a short, rapid drop in CH4 is also present around 1600 CE, which is not present in the WAIS Divide (shown for reference) and Skytrain records.

We hope that these new details have reassured the reviewer on the quality of our age distribution/firn filter modelling, and that the additions to the manuscript ensure other readers have a clearer description to utilise. In answer to the overview question 'A key question is how an ice core with an accumulation of only 13.5 cm/yr can retain atmospheric composition variations that can be used to verify a rapid variation observed in the higher accumulation rate ice from Law Dome (DSS, 60 cm/yr)?'; our extensive smoothing experiments indicate that both the Skytrain and WAIS Divide cores are capable of recording high resolution CO_2 changes and in particular the '1610' atmospheric CO_2 variations, and we have now demonstrated that these experiments are based on accurate age distributions.

Buizert, C. The influence of firn air transport processes and radio-carbon production on gas records from polar firn and ice. (University of Copenhagen, 2011).

Buizert, C. et al. Gas transport in firn: Multiple-tracer characterisation and model intercomparison for NEEM, Northern Greenland. Atmos Chem Phys 12, (2012).

Mitchell, L. E. et al. Observing and modelling the influence of layering on bubble trapping in polar firn. J Geophys Res 120, (2015).

Rosen, J. et al. An ice core record of near-synchronous global climate changes at the Bølling transition. Nat Geosci 7, (2014).

Rubino, M. et al. Low atmospheric CO2 levels during the Little Ice Age due to cooling-induced terrestrial uptake. Nat Geosci 9, (2016).

Validation of ice core air age distributions is useful to quantify the effect of enclosure smoothing on atmospheric variations. One approach is to test the air age distributions derived for the air in ice core bubbles against rapid atmospheric changes of gas species with independently found histories. For example, 14CO2 measurements of Law Dome ice core and firn air, including the DSS ice core, were compared with measurements of the "bomb-pulse" 14CO2 in the atmosphere smoothed by

the model of firn diffusion and bubble close-off (Smith et al., NIMB, 2000). Have measurements of any gas species other than CO2 or CH4 been used to check the Skytrain ice core air age distributions?

Further gas species have not been measured in this section of the Skytrain core, however our continuous CH_4 record was measured specifically to test the compatibility of our CO_2 findings on an independent, high-resolution gas species. Further analysis was outside the scope of this study.

King et al. test whether the smoothing required to remove all of the 1610 CO2 minimum signal is compatible with the CH4 measurements (from line 149). This makes good sense as there are CH4 data through these periods for the 3 cores and CH4 is generally reliably enclosed and stored in air in ice. However, this comparison depends on how well constrained are the WAIS and Skytrain age distributions ("firn filters"). If they are trained on matching the CO2 datasets would they be potentially in error due to the variability that both cores' CO2 measurements show especially in the 1600s?

We note that the firn-filters - the starting point for our enhanced smoothing experiments - are not trained on data, they are purely the consequence of our current, best knowledge of firn smoothing. There is thus no circular logic regarding our starting point for the firn smoothing experiments. To answer this, as above, we have improved our descriptions of the production of our firn filters and added to the discussion on the accuracy of our filters.

It's also difficult to see how well the increased smoothing functions fit the data (Fig 3B). These are quite radical increases in smoothing and it would help to see the CO2 data and their uncertainties plotted alongside.

To clarify this process, we have now added the below description to the supplement alongside FigureR3B (subsequently Figure S1).

To choose the correct level of increased smoothing in our log-logistic distributions (i.e. those that best fit the 'no 1610 dip' scenarios of the Skytrain and WAIS Divide ice core CO_2 records) we generated a wide range of log-logistic functions and then calculated the offsets to the Skytrain and WAIS Divide CO_2 splines for each function, looking for the closest fit. The offset was calculated over a specific time window covering the CO_2 drop. The average offset for each spline and function is then plotted to find the optimal functions, where the offset is lowest (Figure R3B). The different functions are represented by their 'a' value, which is the parameter of the log-logistic function that controls the width of the function and therefore the degree of smoothing. For Skytrain, the three best-fit functions, representing a range of options since none are perfect, were a75, a80 and a85. For WAIS Divide the best-fit functions were a70, a75 and a80, a close agreement. There is little difference between these two options, so we chose to take forward the range for Skytrain based on our later experiments being used to investigate other Skytrain gas records specifically.

We refer to this in both our main manuscript *('We increase the width of the smoothing until the convolution output best matches the measured CO_2 records of WAIS Divide and Skytrain using a test which calculates the offset between the convolutions and datasets (See supplement and Figure S1')*) and our log-logistic function methods section *('Three filters are chosen which best reproduce these records, which were the three filters calculated to have the lowest offset to the CO2 splines of WAIS Divide and Skytrain (see Supplement Figure S1)).*

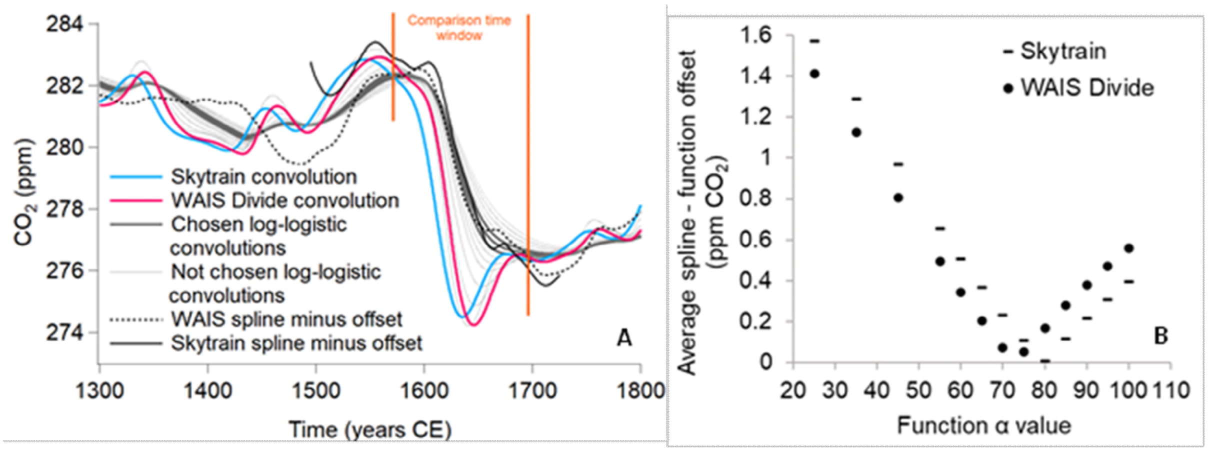

Figure R3B: Figure illustrating the choice of log-logistic functions which best recreate the Skytrain and WAIS Divide CO_2 records (i.e. which eliminate the '1610 dip' when convolved with the Law Dome CO_2 record). Each log-logistic function convolution (A) was compared to the Skytrain and WAIS Divide CO_2 splines over a time window covering the entire CO_2 drop into the Little Ice Age by calculating the CO_2 offset of each at annual resolution. The Skytrain and WAIS divide splines have been adjusted by a consistent absolute CO_2 value to plot over the convolutions since the latter are based on the absolute values of Law Dome. The average offset is plotted (B) to indicate the functions which best fit the splines, where the offset is lowest.

The CH4 data could also be shown in Fig 3C as well as the spline.

The unsmoothed continuous Skytrain CH_4 data are plotted in Figure S2, and we have updated the caption of Figure 3 to reference this directly: *'For reference, the full Skytrain continuous CH_4 record is shown in Figure S2.'* The Skytrain data was continuously measured using CFA analysis, thus is presented in a smoothed spline form on the main manuscript plot for best comparability to the discrete measurements of Law Dome and WAIS and so as not to overfill the plot.

It should be noted that there are rapid CH4 variations in Law Dome DSS that are not observed in either Skytrain or WAIS, curiously also around 1600AD. It would be unlikely that these CH4 differences were caused by the same artefact that is proposed to have affected CO2.

It is indeed interesting that the CH_4 record of Law Dome shows a rapid variation at 1600 which is not captured by either WAIS Divide or Skytrain. As in response to an earlier comment, we have now added discussion of the rapid CH_4 variations to the manuscript:

'In an alternative approach, one could use the CH_4 records from all three cores to determine age distributions of WAIS Divide and Skytrain independently of the CO_2 record and provide not only estimates of the upper limits of the firn age widths (as done here), but also the lower limits. However, this would require high-resolution, continuous CH_4 data from Law Dome. Tentatively, we can place some constraints on the lower limit of the firn age distributions by examining a CH4 minimum that immediately precedes the CO_2 drop (~1590 CE). This minimum is fortuitously nearly coincident with the CO_2 drop of interest (Figure S4 (or R3B here), about half the width of the CO_2 drop (~20 years versus ~40 years), and about the same width as the gas age distribution of Skytrain and WAIS Divide. If our original estimates of smoothing based on firn models were accurate, we would predict that CH_4 minimum would be absent in WAIS Divide and Skytrain. This is clearly the case in the WAIS Divide record and most likely the case in the Skytrain record (although our measurement unfortunately did not extend to the predicted levelling off in CH_4 post 1600 CE (Figure 3). However, other features present in the low-resolution Law Dome data are not obviously comparable to the WAIS and Skytrain

features such as divergence in the preceding CH_4 bump of the 1500s CE. Measurements of CH_4 and other gases at the Law Dome site via continuous flow analysis (CH_4 CFA publications) are thus a crucial prerequisite for future work on understanding the smoothing imparted on ice core gas records by firn processes.'

Due to the large differences in data resolution between each of the three CH_4 records, it is difficult to give a precise enough comparison between records to comment on artefacts. Future work in high resolution CH_4 analysis in cores for this time period, as suggested, would be needed.

The general approach of King et al. is similar in many ways to that used by Rubino et al., Nature Geo 2016, who compared the CO2 changes in the WAIS and DML (also lower accumulation than Law Dome) ice cores with those in the Law Dome DSS core. The CO2 measurements were consistent with the DSS CO2 record convolved through the bubble air age distributions for both those ice cores but neither could confirm or rule out the 1610 minimum in DSS. Without evidence that the Skytrain bubble air age distribution is sufficiently narrow and not broadened by its relatively low accumulation rate, it is difficult to see how it could offer a significant improvement.

Rubino et al. 2016 do indeed follow a similar approach in proposing the use of a third, independent, ice core record of CO_2 through this time to resolve the difference between the existing Law Dome and WAIS Divide records. However, a key difference to this study is that the age distribution of the core they utilise, DML, is significantly broader, and thus the record significantly more smoothed, than that of Skytrain, the core used in this study. Thus, we present a record which has the capability of recording the '1610 dip', while the Rubino study acknowledges themselves that the DML record would not have the capability to record this feature. As in our earlier responses, we have improved the description of the generation of our age distributions/firn filters and thus hope to have answered the doubts around the capability of the Skytrain record to record the '1610 dip'. Thus, we can reliably compare the various age distributions to highlight the improvement in comparison to DML of our record; the age distribution width of Law Dome is lowest, at 8 years, with both WAIS and Skytrain having similar distributions at 19 and 23 years, respectively. The age distribution of DML is three times higher than this, at 65 years. This would increase the smoothing significantly. Indeed, the DML distribution is wider than the Law Dome '1610 CO_2 drop' in its entirety, and thus would not capture such an event in its record even if it were a real atmospheric signal. In contrast, as our extensive smoothing investigations demonstrate, while the WAIS Divide and Skytrain distributions are not as high in resolution as Law Dome, they are still at a high enough resolution that they would faithfully capture the 1610 drop if it were a true atmospheric signal. With our improved justification that our Skytrain age distribution is accurate, this holds true. Additional robustness to this conclusion is added by demonstrating that this holds true in both the WAIS Divide and Skytrain cores.

A final general comment regards the comparison of the Law Dome 1610 minimum with carbon fluxes from land use change scenarios. The authors interpret the inability of land use models to account for the measured 1610 minimum as evidence that it is implausibly large. It is interesting to make model-data comparisons like these but I think the land use estimates for that time could also be uncertain.

We certainly agree on the uncertainty with land use estimates, and we discuss this in the text: *'It is important to note that underlying the state-of-the art land use and carbon cycle models are based on population estimates that are very coarse resolution (100 years until 1700 CE). Now that our new data precisely define the timing and magnitude of carbon uptake, further work modelling population*

dynamics, land-use change and carbon cycle changes during the New World Pandemics is warranted.'

More importantly, this claim seems to ignore the CO2, 13CO2 and COS measurements and modelling presented by Rubino et al. (Nature Geo., 2016) as evidence for a climate role in the Little Ice Age CO2 drop (~1600-1800).

We have added this citation to the beginning of the existing discussion on potential climatic influence at 1610 CE: *'Alternatively, the CO_2 decrease could be due in some part to natural carbon cycle feedbacks that were triggered by a cooling in the Northern Hemisphere [5].'*

Arguing that a low accumulation ice core can better record rapid atmospheric CO2 variations than a higher accumulation counterpart requires convincing demonstration that the air age distribution has been fully accounted for and that there are no artefacts in the ice core measurements- of both high and low accumulation cores. King et al. go some of the way in doing this for their Skytrain study but more is required to make their claims more robust.

In summary for the more detailed responses above, we have improved the reporting of how our age distributions were generated and demonstrated their accuracy, and combined with the response to Reviewer 1, have improved the error analysis on our CO_2 data, to report variability in the records more realistically.

Given the importance of ensuring that past CO2 variations are accurately reconstructed from ice cores I hope that they can make the suggested improvements. Their final statement that new very high accumulation (air age resolution) Antarctic ice cores are needed must stand as a high priority.

We appreciate the support of the reviewer in moving this study forward to publication. A new high-resolution core would be an exciting move forward in the field which we hope this study can help to motivate.

Other, specific notes on the manuscript:

Lines 45, 46, 53, 55. Please say whether and how bubble close off is accounted for in these estimates, and later on in the analysis.

We have improved our description of firn filter modelling, as detailed above, throughout the manuscript.

Line 56. 0-6 ppm is given in Ahn et al.

Changed to *'range from 0-6 ppm higher….'.*

Line 60. Is this saying that both records could have artefacts?

This line refers to the WAIS Divide values being elevated, and has been rephrased to more clearly relate to this: *'the absolute mixing ratios of WAIS Divide are ~2-4 ppm higher than those of Law Dome between 750 - 1800 CE [3,4], though the exact cause of this artefact remains elusive.'*

Line 68, 77. Should also say that the other interpretation is climate-CO2 feedback.

This has been added: *'…,as well as inferred climate-CO_2 feedback'* with reference to the work of Rubino 2016 as mentioned by the reviewer above.

Line 80. Prefer "interpretation" or "understanding" to accounting.

Changed to '*interpretation'.*

Line 94. More information on Skytrain relevant to ice core CO2 would be helpful: temperature, melt events, age and uncertainty, locations, how drilled…. Either here or in Methods/Supplementary.

Site temperature has been added to the existing details on age scale and accumulation rates given in the methods. Details of the drilling of the Skytrain ice core and the site characteristics have been previously published in reference [32], while the details of chemistry and dating in the top 2000 years of the core have been published in [33], and the gas age scale for the entire core published in [34]. Each of these are referenced in the methods section alongside some overview data for each, however a sentence has been added to clarify: *'For further detailed information on drilling, site characteristics and age scale generation please see references [32,33,34].'*

Line 100. The first part of the CO2 decrease actually looks comparable to Law Dome.

In this section we give values that consider the total CO_2 decline in each core for the entire 'CO_2 drop'. It is correct that within this decline there may be very shorter, decadal-scale, periods where rates of change agree with both WAIS Divide and Law Dome. However, this is tricky to compare in a robust way due to differences in sample resolution between cores. The main focus for this manuscript is the comparison in the overall change between cores.

Line 109. As in general comments above, regions of large variability in both Skytrain and WAIS.

Please see earlier detailed discussion on additional error/variability analysis.

Line 117. Low pass filtering occurs in firn and bubble close of region.

Added *'...and during bubble close off'.*

Line 119. Are all air age distributions using the same statistic?

Yes, all distributions are given at Full Width Half Maximum (FWHM). The first distribution presented in the manuscript has been changed to *'(gas age distribution width of 8 years at Full Width Half Maximum (FWHM)'* and all others follow the style of *'(gas age distribution width of 19 years FWHM)'.*

Line 122. For clarification: …(about 40 years) was ana actually atmospheric CO2 change recorded in Law Dome ice but smoothed away in both Skytrain and WAIS?...

This has been added: *'...CE (about 40 years) was an atmospheric CO_2 change that was recorded at Law Dome…'.*

Line 128. "Firn filters" are also referred to as Greens Functions for the purpose of convolutions and forward and inverse modelling signals.

Added to the methods section on firn filter modelling: *'These may also be referred to as age distributions and/or Green's Functions in similar studies.'*

Line 131, 132. The convolved minimums are much more subtle than the originals, and are actually not much different from the data, especially Skytrain, given the stated uncertainties and the additional variability.

The reviewer correctly highlights that it is difficult to compare the new convolved histories to the original data in the existing figure, which we believe is due to both the offsets and the faded colour of the original data in the background. If the offsets are removed, as in Figure R3D below, it becomes

evident that the convolved histories are recreating much of the dip in the Law Dome spline. In the figure, we feel it better to maintain the offsets to allow close comparison to the original datasets. We have therefore adapted the figure in the manuscript by making the splines of the original data bold, rather than faded, as Figure R3E below, so that a clearer comparison can be seen. We have also now included the updated spline confidence intervals on the plot, with the increased capture of variability, so that it can be observed how the convolutions are differentiated from the original records by being beyond the scope of variability in the datasets.

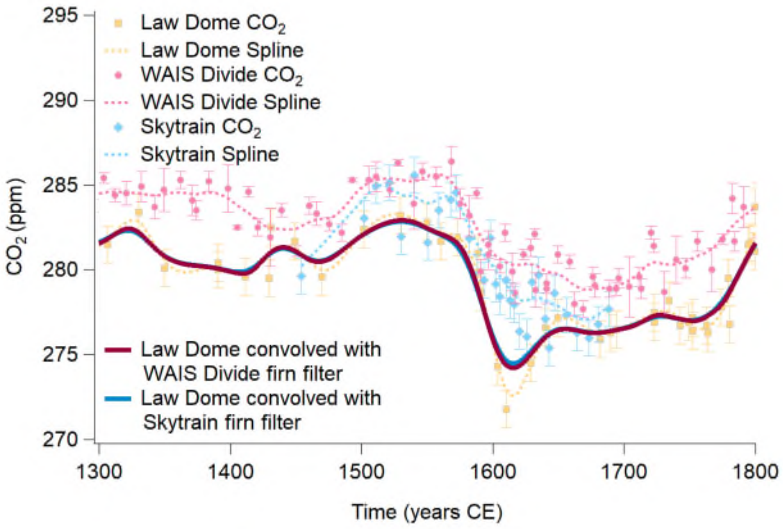

Figure R3D: Convolved histories overlying original CO_2 records, as in manuscript Figure 2, but with offset adjustments removed.

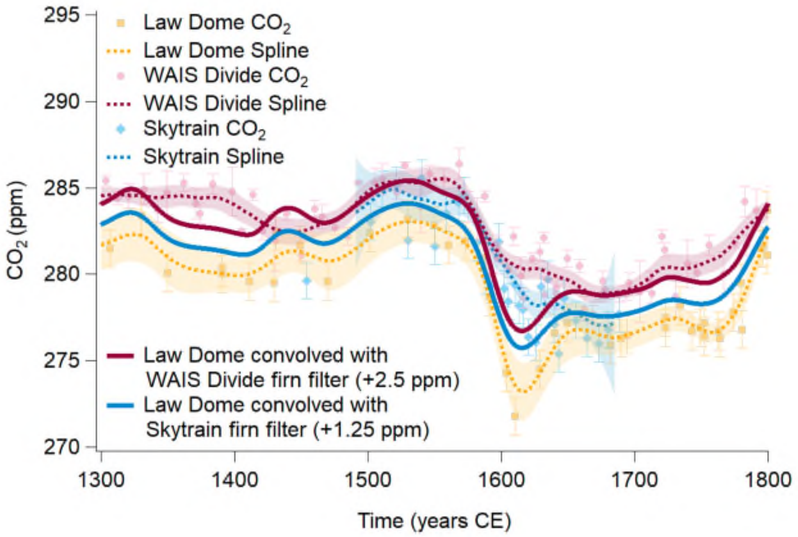

Figure R3E: Convolved histories overlying original CO_2 records, as in manuscript Figure 2, but with smoothing splines of the original CO_2 records in bold to allow easier comparison between these and the convolved histories, and with additional confidence intervals of the splines. Figure R3C has replaced Figure 2A in the manuscript.

Line 138. Again, what is included in the firn filters?

Please see earlier discussion on firn filters for more detail.

Line 150. If it is a true signal and if it is enclosed and preserved without unexpected changes…

Added *'…if it is a true paleo-atmospheric signal, assuming no unexpected changes have occurred, but is this robust…'*

Line 158. Could the Skytrain CH4 data be plotted please?

The Skytrain continuous CH_4 data is plotted in Figure S2, and we have updated the caption of Figure 3 to reference this directly*: 'For reference, the full Skytrain continuous CH_4 record is shown in Figure S2.'* The Skytrain data was continuously measured using CFA analysis, thus is presented in a smoothed spline form on the main manuscript plot for best comparability to the discrete measurements of Law Dome and WAIS and so as not to overfill the plot.

Figure 3B. Could the CO2 data be plotted to see how well the convolutions fit?

As in our earlier response, we have now added a new section and figure to the supplement to improve the description of how we chose our increased smoothing functions and convolutions, which plots the CO_2 data alongside the convolutions. This new figure allows this to be seen more clearly than if we were to also plot it on Figure 3B as the latter figure is very small and becomes difficult to read with further lines plotted.

Figure 3C. Please show Skytrain data points.

The Skytrain CH_4 data is plotted in Figure S2, and we have updated the caption of Figure 3 to reference this directly: *'For reference, the full Skytrain continuous CH_4 record is shown in Figure S2.'.* The Skytrain data was continuously measured using CFA analysis, thus is presented in a smoothed spline form on the main manuscript plot for best comparability to the discrete measurements of Law Dome and WAIS Divide and so as not to overfill the plot.

Figure 4. It would be help if the ocean-atmosphere fluxes were shown or at least mentioned.

Thank you for the suggestion. We have included the ocean-atmosphere fluxes in a supplemental figure (as Figure R3E below, or therein Figure S5) along with a comparison to previously published results to show the good agreement between the OSU box model (this study) and previous work using the HILDA model (Bauska et al., 2015). This is referenced in the manuscript: *'The ocean-atmosphere carbon flux (Figure S5) is allowed to evolve freely…'*

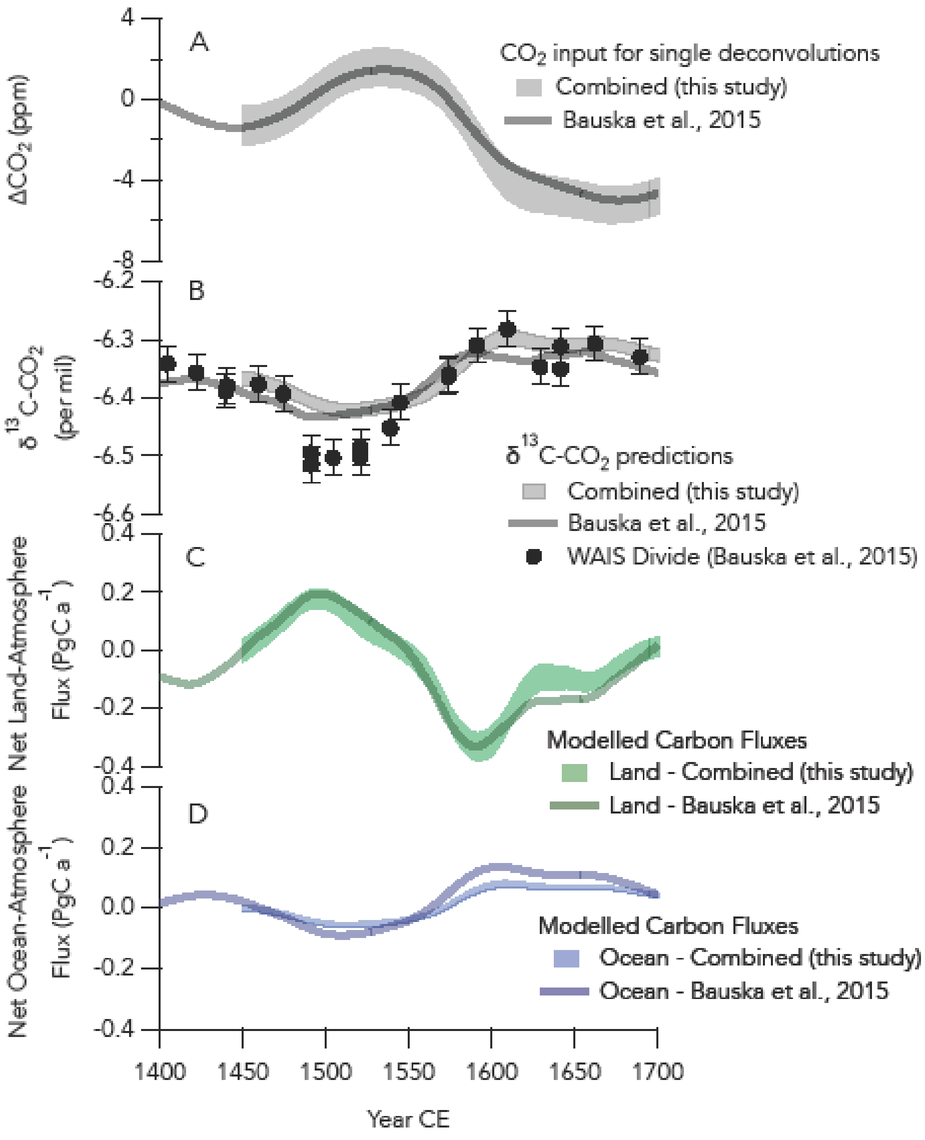

Figure R3F: A comparison of the land and ocean carbon fluxes predicted in this study (shaded bands) with the OSU Carbon Cycle Model and a previous study using just the WAIS Divide ice core data and the HILDA model (Bauska et al. 2015) (darker lines). All experiments are single deconvolution experiments and thus use only the atmospheric CO_2 data (A) to predict the land-atmospheric carbon flux (C). The atmospheric d13C-CO_2 (B) and ocean-atmosphere fluxes (D) are allowed to freely evolve in the model. The single deconvolutions fail to capture the d13C-CO_2 minimum that precedes the 1610 drop but do capture the broader maximum within the top itself.

Bauska, T. K. et al. Links between atmospheric carbon dioxide, the land carbon reservoir and climate over the past millennium. Nat Geosci 8, (2015).

Line 161. Given the points raised above this would be better stated as “a plausible”.

Changed to ‘*plausible*’.

Line 232. Also as stated above, there are other possible reasons for the CO2 change.

We have added the citation of Rubino et al. 2016 as suggested earlier: *'Alternatively, the CO_2 decrease could be due in some part to natural carbon cycle feedbacks that were triggered by a cooling in the Northern Hemisphere [5].'*

Line 261, 262. Based on the reasons given above, this statement is possibly too confident. Suggest removing the word "strong" unless more evidence as mentioned earlier can be shown.

We have made all suggested revisions to show that our data and analysis is of high and reliable quality, thus we do not completely remove the wording here but do change 'strong' to *'robust'*.

Line 292. What is the range/uncertainty of the CH4 blank?

Added: *'An average blank correction of 8.3 ± 2.7 SD ppb…'*

Section at line 355. Please be clear about what processes are included in the firn filter modelling, especially bubble close off and how it interacts with the diffusion in the firn column. This is crucial to the resulting air age spread and the conclusions of this work.

We have improved our description of firn filter modelling, and indicated references to other published work reporting further detail on the model validation and the detailed parameterisation which is outside the reporting scope of this study.

Reviewer #1 (Remarks to the Author):

I'm happy with the revisions and have only one concern which is easily addressed by minor wording changes. This and a few other minor suggestions are detailed below. The concern relates to the claim that the Law Dome dip in CO_2 around 1610 is incompatible with land use scenarios.

Specific points:
26-29. Needs rewording because it doesn't recognize that the Law Dome dip might have had a cause other than land use. I think the valid point is that the more gradual CO_2 decrease seen in Skytrain and WAIS is easily explained by CO_2 uptake driven land-use following new-world old-world contact and helps valid some land-use reconstructions. In contrast, the Law Dome dip, if real, requires some unknown process.
82. I'd expect references to the Skytrain core (32, 33) here.
268-270. Needs rewording for the same reason as above.
302 I'd cut "robust". Adding the word "robust" doesn't add robustness.This study is an important advance and strongly motivates further work. But it is not the last word.
307. Needs rewording for the same reason as above.
Figure 3. I urge that the actual Skytrain CH_4 dataset be shown in Figure 3, and not just the spline. Perhaps the authors were concerned this would clutter the figure? This could be easily addressed by using a faint color for the actual data.

Reviewer #3 (Remarks to the Author):

Second review, "Reconciling ice core CO_2 and land-use change following New World-Old World contact"
King, A.C.F. et al., resubmitted to Nature Communications.
December 2023

Thank you to the editor of Nature Communications for the opportunity to review this resubmission and to the authors for the considered comments they have made to both reviews and their careful revisions. I think the manuscript has improved significantly.
My main concern remains with the quantification of the ice core air age distributions, or at least their uncertainties, and how that may impact the confidence of the conclusions. The new paragraph (line 181) says that tuning age distribution widths using CH_4 records would require higher resolution CH_4 data from Law Dome (the same also applies to the WAIS CH_4 data which have low sampling resolution) from CFA measurements of CH_4 and other gases. Indeed this is one limit of intercomparing ice core gas records. The use of independent atmospheric gas measurements is preferred for tuning air age distributions of ice core gas records (not just firn air records), as was done for Law Dome using $^{14}CO_2$ and mentioned in my first review. The authors note in their rebuttal that, for the Skytrain and WAIS cores, "We note that the firn-filters - the starting point for our enhanced smoothing experiments - are not trained on data…". Given that a main thrust of this manuscript relies on accurately quantified air age distributions I think this point should be made in the manuscript, as it is an important difference between the records. Together with the observation that WAIS, and less so Skytrain, have relatively elevated CO_2 concentrations, and the variability within each of the ice core CO_2 records, I think there are good reasons to reword the multiple claims about the Law Dome CO_2 being (the only?) artefact data during the 1610 event (lines 24, 177, 178, 301).
The abstract of the revised version appears unchanged. I think it should be updated to include some of the changes made to the manuscript following the points raised in the reviews. In particular, saying that the sudden 1610 CE decrease is most likely an artefact, seems too strong given the potential uncertainties in all of the ice core records discussed in the manuscript. Also, the decrease at 1610 being too rapid to be explained by land use change should be accompanied by the point in the review that a climate influence on terrestrial carbon is also compatible with lower CO_2 as well as the previously published evidence of $^{13}CO_2$, COS and CH_4 changes. This last point is important because much of the focus on the 1610 CO_2 minimum of this manuscript and some of the publications it cites is on the event being ascribed to an anthropogenic cause and a potential marker of the Anthropocene (the Orbis spike), whereas a climatic cause is also possible.

REVIEWERS' COMMENTS

Reviewer #1 (Remarks to the Author):

I'm happy with the revisions and have only one concern which is easily addressed by minor wording changes. This and a few other minor suggestions are detailed below. The concern relates to the claim that the Law Dome dip in CO2 around 1610 is incompatible with land use scenarios.

Thank you to the reviewer for their time in reviewing our corrections and for their positive comments.

Specific points:

26-29. Needs rewording because it doesn't recognize that the Law Dome dip might have had a cause other than land use. I think the valid point is that the more gradual CO2 decrease seen in Skytrain and WAIS is easily explained by CO2 uptake driven land-use following new-world old-world contact and helps valid some land-use reconstructions. In contrast, the Law Dome dip, if real, requires some unknown process.

We agree that there are alternative explanations possible for driving changes in atmospheric CO2 outside the land-use scenarios that are the focus of this study. Our focus in this study has been to reconcile the different land use change scenarios available with the ice core records, only. This is because the land use scenarios have been presented and debated in a number of studies, whereas the alternative natural drivers are currently much more uncertain. Indeed, outside of our call for improved ice core gas records in the Common Era, made in our concluding remarks, we also discuss the need for further modelling work at the end of our section on land use change; 'Now that our data precisely define the timing and magnitude of carbon uptake, further work modelling population dynamics, land-use change and carbon cycle changes during the New World Pandemics is warranted'.

In our paper, we already offer discussion on alternative possible scenarios in the section beginning; 'Alternatively, the CO2 decrease could be due in some part to natural carbon cycle feedbacks that were triggered by a cooling in the Northern Hemisphere[5]. In the case of the 1610 CE CO2 minimum this would require a sudden cooling and similarly rapid warming…'.

We revisit the idea of alternative possibilities in our closing remarks of the paper. 'Whether the CO2 decrease resulted from natural or anthropogenic processes remains an open question'.

To expand this further in response to the reviewer requests, we have added:

'Whether the CO2 decrease resulted from natural or anthropogenic processes, *or a combination of the two*, remains an open question. *It is possible that large-scale land abandoned, in tandem with a hitherto unidentified climate-carbon feedback, could explain the minimum in the Law Dome record.'*

In our abstract, we state that we are considering land use change in the Americas specifically, and discuss whether ice core data fits these land use scenarios. We prefer not to add additional discussion of all alternative possibilities in to our abstract since these are more uncertain and are not a focus of our paper.

82. I'd expect references to the Skytrain core (32, 33) here.

These references have been added.

268-270. Needs rewording for the same reason as above.

As above, we have added further discussion to our closing remarks of the paper to emphasise possible alternative scenarios in carbon cycle drivers to the Law Dome 1610 CO2 minimum. Where we mention land use change scenarios throughout the rest of the paper, we are always clear to state that they are land use change scenarios only, and we prefer not to add the same caveat of possible alternative explanations into each stage of the discussion to avoid repetition, and because these possible alternative scenarios were not the focus of the study.

302 I'd cut "robust". Adding the word "robust" doesn't add robustness. This study is an important advance and strongly motivates further work. But it is not the last word.

This word has been removed.

307. Needs rewording for the same reason as above.

As above, we have added further discussion to our closing remarks of the paper to emphasise possible alternative scenarios in carbon cycle drivers to the Law Dome 1610 CO2 minimum. Where we mention land use change scenarios throughout the rest of the paper, we are always clear to state that they are land use change scenarios only, and we prefer not to add the same caveat of possible alternative explanations in to each stage of the discussion to avoid repetition, and because these possible alternative scenarios were not the focus of the study.

Figure 3. I urge that the actual Skytrain CH4 dataset be shown in Figure 3, and not just the spline. Perhaps the authors were concerned this would clutter the figure? This could be easily addressed by using a faint color for the actual data.

This dataset has been included in the figure.

Reviewer #3 (Remarks to the Author):

Thank you to the editor of Nature Communications for the opportunity to review this resubmission and to the authors for the considered comments they have made to both reviews and their careful revisions. I think the manuscript has improved significantly.

Thank you to the reviewer for their time in reviewing our corrections and for their positive comments.

My main concern remains with the quantification of the ice core air age distributions, or at least their uncertainties, and how that may impact the confidence of the conclusions. The new paragraph (line 181) says that tuning age distribution widths using CH4 records would require higher resolution CH4 data from Law Dome (the same also applies to the WAIS CH4 data which have low sampling resolution) from CFA measurements of CH4 and other gases. Indeed this is one limit of intercomparing ice core gas records. The use of independent atmospheric gas measurements is preferred for tuning air age distributions of ice core gas records (not just firn air records), as was done for Law Dome using 14CO2 and mentioned in my first review. The authors note in their rebuttal that, for the Skytrain and WAIS cores, "We note that the firn-filters - the starting point for our enhanced smoothing experiments - are not trained on data…". Given that a main thrust of this manuscript relies on accurately quantified air age distributions I think this point should be made in the manuscript, as it is an important difference between the records.

We have added the requested information into the Firn Filter modelling sub-section of our Methods section in the main manuscript, as *'We note that the firn-filters - the starting point for our enhanced smoothing experiments - are not trained on data, they are purely the consequence of our current, best knowledge of firn smoothing.'* This follows introduction of the firn filter model and precedes

description of our validation of the parameters used, which are based on our firn smoothing knowledge.

Together with the observation that WAIS, and less so Skytrain, have relatively elevated CO2 concentrations, and the variability within each of the ice core CO2 records, I think there are good reasons to reword the multiple claims about the Law Dome CO2 being (the only?) artefact data during the 1610 event (lines 24, 177, 178, 301).

In the abstract, we specifically refer to a 'small number of anomalously low values', to emphasise that we are referring to a specific feature in the Law Dome record, the 1610 CE CO2 minimum, and not the Law Dome record more widely. Unfortunately, we cannot comment on the wider offsets between records without further data, i.e. the idea that WAIS Divide values are higher due to data artefacts, without more data, which we discuss in the manuscript already.

Where we refer to the artefact data in other locations in the manuscript, we have toned down the language to incorporate the ideas of these wider offsets and to refer again most specifically to the 1610 CO2 feature itself:

'We therefore conclude that no plausible smoothing scenarios exist to account for the absence of the 1610 CE CO2 minimum in the WAIS Divide and Skytrain CO2 records, and ~~the only~~ *a* plausible explanation is that the Law Dome 1610 CE CO2 minimum is *formed of a small number of* artefact data *points*'.

And, as below, in the abstract we have changed the wording from 'most likely an artefact' to *'may be an artefact'.*

The abstract of the revised version appears unchanged. I think it should be updated to include some of the changes made to the manuscript following the points raised in the reviews. In particular, saying that the sudden 1610 CE decrease is most likely an artefact, seems too strong given the potential uncertainties in all of the ice core records discussed in the manuscript.

We have changed the wording from 'most likely an artefact' to *'may be an artefact'.*

Also, the decrease at 1610 being too rapid to be explained by land use change should be accompanied by the point in the review that a climate influence on terrestrial carbon is also compatible with lower CO2 as well as the previously published evidence of 13CO2, COS and CH4 changes. This last point is important because much of the focus on the 1610 CO2 minimum of this manuscript and some of the publications it cites is on the event being ascribed to an anthropogenic cause and a potential marker of the Anthropocene (the Orbis spike), whereas a climatic cause is also possible.

Please see responses to Reviewer 1, where we describe how we consider alternative scenarios to land use change in driving atmospheric CO2 changes, where we have added to this description, and where we emphasise that the focus of this paper is only in reconciling the land use change scenarios to ice core data.
